# Angiostatic cues from the matrix: Endothelial cell autophagy meets hyaluronan biology

**DOI:** 10.1074/jbc.REV120.014391

**Published:** 2021-01-13

**Authors:** Carolyn G. Chen, Renato V. Iozzo

**Affiliations:** Translational Cellular Oncology Program, Sidney Kimmel Cancer Center, Sidney Kimmel Medical College at Thomas Jefferson University, Philadelphia, Pennsylvania, USA

**Keywords:** AMP-activated kinase (AMPK), cell signaling, proteoglycan, perlecan, decorin, extracellular matrix, autophagy, angiogenesis, hyaluronan, endothelial cell, hyaluronan synthase 2, vascular biology

## Abstract

The extracellular matrix encompasses a reservoir of bioactive macromolecules that modulates a cornucopia of biological functions. A prominent body of work posits matrix constituents as master regulators of autophagy and angiogenesis and provides molecular insight into how these two processes are coordinated. Here, we review current understanding of the molecular mechanisms underlying hyaluronan and HAS2 regulation and the role of soluble proteoglycan in affecting autophagy and angiogenesis. Specifically, we assess the role of proteoglycan-evoked autophagy in regulating angiogenesis via the HAS2-hyaluronan axis and ATG9A, a novel HAS2 binding partner. We discuss extracellular hyaluronan biology and the post-transcriptional and post-translational modifications that regulate its main synthesizer, HAS2. We highlight the emerging group of proteoglycans that utilize outside-in signaling to modulate autophagy and angiogenesis in cancer microenvironments and thoroughly review the most up-to-date understanding of endorepellin signaling in vascular endothelia, providing insight into the temporal complexities involved.

The extracellular matrix (ECM) consists of a three-dimensional structural scaffold of macromolecules that provides an extensive reservoir of complex signaling molecules, masterfully orchestrating a plethora of biological functions affecting surrounding cells and tissue (1). Among the major macromolecules in the ECM are glycosaminoglycans (GAGs), proteoglycans (PGs), glycoproteins, proteinases, collagens, laminins, fibronectin, and elastin. Often referred to as the “outside-in” cues of the matrix, bioactive extracellular molecules are tightly regulated to bind to receptors such as integrins, certain PGs, CD44, discoidin domain receptors, innate immune receptors, and receptor tyrosine kinases (RTKs) on the cell surface that initiate specific paracrine and/or autocrine intracellular signaling within the cell (2, 3, 4, 5, 6, 7, 8, 9, 10, 11, 12, 13, 14, 15). Further, the ECM also serves as a reservoir for signaling molecules, such as growth factors and cytokines, as these can bind to specific ECM molecules and are later liberated to bind to their cognate receptors. For example, a variety of signaling effectors bind to heparan sulfate (HS) chains that are later disseminated upon heparanase activity. Matrix metalloproteases function similarly, liberating bound growth factors and cytokines in the ECM to their respective cell-surface receptors (16). These cues synchronize a remarkable array of cellular functions ranging from tissue homeostasis to proliferation, cell migration, wound healing, survival, and development (17, 18, 19).

Here, we review a new and emerging area of ECM biology in which certain PGs and bioactive PG fragments regulate autophagy and angiogenesis (20, 21). We highlight a novel, autophagy-dependent regulation of angiogenesis involving hyaluronan (HA) and hyaluronan synthase 2 (HAS2). This is of increasing importance as PG-induced autophagy and angiogenesis show functional implications in cancer progression. We begin with a general overview of autophagy and its implications in cancer progression. Next, we discuss HA biology and synthesis via HAS2 regulatory mechanisms. We then delve into the major PGs and PG fragments involved, namely endorepellin, decorin, endostatin, biglycan, and lumican, focusing on our current understanding of perlecan and endorepellin signaling. Importantly, we explore HAS2 as a novel link between autophagy and angiogenesis downstream of endorepellin signaling and conclude with open questions and potential implications for future research targeting angiogenesis and autophagy.

## Autophagy

Macroautophagy (hereafter referred to as autophagy) is the evolutionarily conserved catabolic process in which cytoplasmic constituents, including superfluous or damaged proteins, lipids, organelles, and intracellular pathogens, are targeted for degradation via the autophagosome-lysosome (22). Originating as a double-membrane, cup-shaped phagophore, the preautophagosomal structure (PAS) recruits lipid bilayer and autophagy protein complexes, expanding its edges around cytoplasmic molecules targeted for degradation and forming a spherical mature autophagosome. These large intracellular vacuoles are then tethered to the microtubule network and trafficked toward the cell center, where lysosomes reside. Following docking and fusion of the autophagosome to the lysosome, lysosomal hydrolases degrade the autophagosomal inner membrane along with its inner cytoplasmic contents (23, 24, 25). In mammalian autophagy, this intricate process is regulated and coordinated by a host of ∼20 autophagy-related (ATG) proteins (25). The end result of this self-digestion pathway is a recirculation of liberated nucleotides, amino acids, fatty acids, sugars, and ATP that are recycled back into the cell to maintain homeostasis of cell metabolism, survival, and upkeep (22, 26).

Autophagy exerts a strong influence on the pathophysiologies of a multitude of diseases, including neurodegeneration, autoimmune diseases, heart disease, infection, and cancer (25, 27). Specifically, in the cancer cell, autophagy engages in either a pro- or anti-tumorigenic fashion, depending on the stage of carcinogenesis. In the early stages of tumorigenesis, autophagy inhibits tumor initiation, proliferation, and invasion as it suppresses genome mutagenesis, chronic tissue damage, inflammation, cell injury, and the oncogenic aggregation of p62 (28, 29, 30). Furthermore, defective autophagy as demonstrated in genetic deletion of autophagic genes *Becn1* or *Atg7* in mice resulted in spontaneous tumors and malignancy (31, 32, 33). Switching roles entirely in later stages of tumorigenesis during invasion and metastasis, autophagy facilitates tumor growth, metabolism, survival, metastasis, and resistance to therapeutic drugs. Mechanistically, this occurs as autophagy protects viability, proliferation, and homeostatic processes of the cancer cell, protecting the cell against various stresses, such as nutrient deprivation, hypoxia, chemotherapy, DNA damage, and metabolic stress (28, 30, 34, 35, 36, 37).

As with the majority of cancer research, studies investigating the impact of autophagy have predominantly focused on autophagy in the cancer cell itself. Recently, a growing body of work targeting the tumor microenvironment has emerged demonstrating the critical role of certain proteoglycans that regulate autophagy in peritumoral stromal cells and their influences on cancer angiogenesis (38, 39). The majority of this work focuses on these proteoglycans that modulate angiogenesis in stromal cells as opposed to the cancer cell.

Beyond the cytoprotective function of autophagy to maintain intracellular homeostasis in the endothelium, the processes of autophagy and angiogenesis are not generally understood as being linked. The concept highlighted in this work of inhibiting angiogenesis in vascular endothelial cells via protracted autophagy has only been explored within the context of matrix-derived PG signaling. In this review, we further discuss the integration of HA synthesis as a novel mechanistic link connecting these two processes.

## Hyaluronan biology

**Figure 1:**
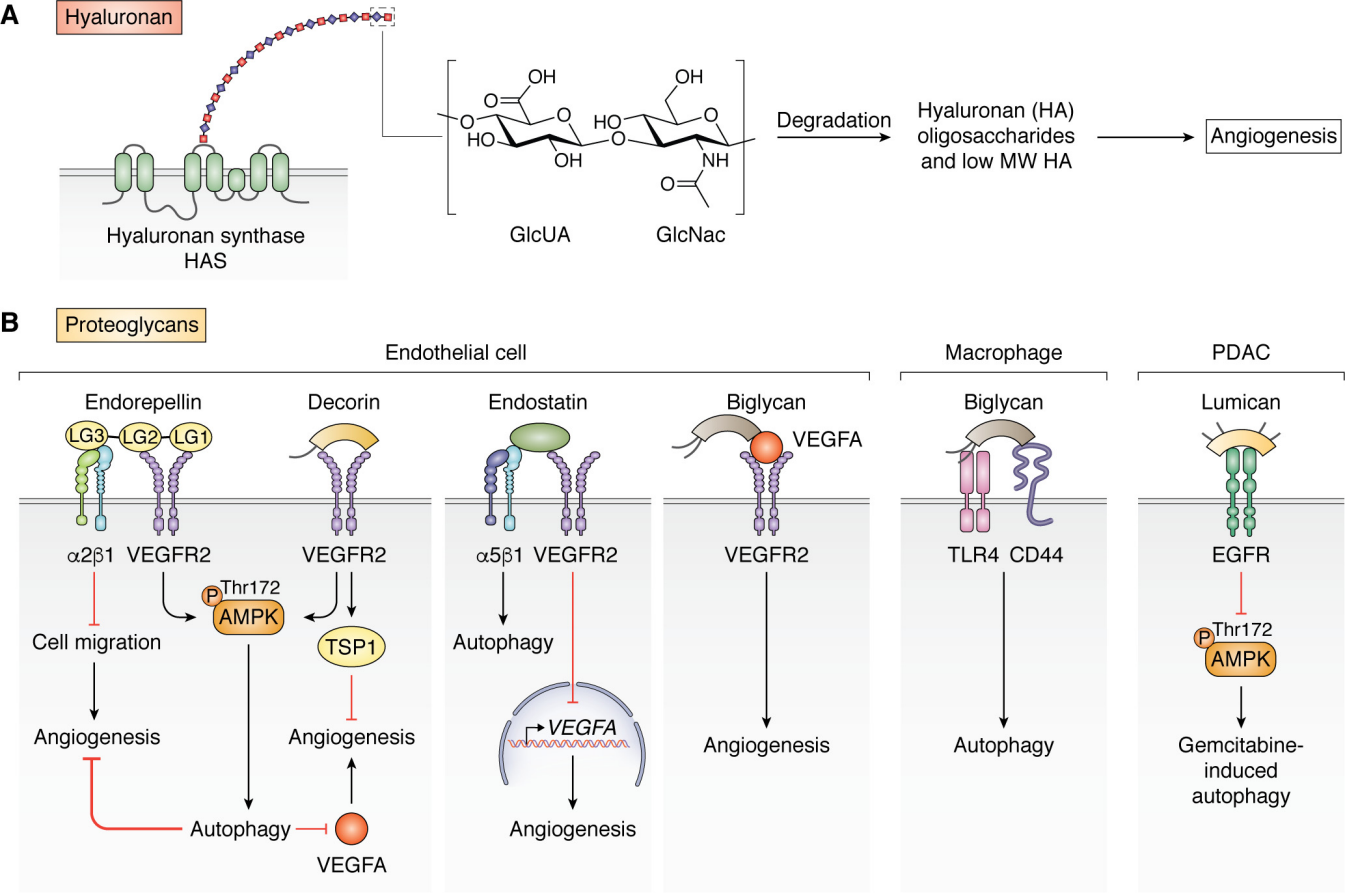
**ECM signaling in autophagy and angiogenesis.***A*, an overview of cell-surface proteoglycan signaling of endorepellin, decorin, endostatin, biglycan, and lumican resulting in modulations on autophagy and/or angiogenesis. Cell types range from endothelial cells to macrophages to pancreatic ductal adenocarcinoma (PDAC). *B*, summary of pro-angiogenic HA signaling starting with synthesis of HA from GlcUA and GlcNAc by HASs at the plasma membrane and ending with the subsequent degradation of HMW HA into LMW polysaccharides.

A key player in ECM biology, HA is a ubiquitously expressed and predominant GAG found in all tissues and body fluids, regulating a complex network of functions (40). Although sophisticated in action, HA intrinsically possesses a physical structure that is astonishingly simple, composed of linear repeating disaccharide units of GlcNAc and GlcUA linked by β-(1,3) and β-(1,4) glycosidic bonds (Fig. 1*A*) (40). It uniquely exists as the only nonsulfated GAG and does not covalently bind to a protein core. Physiologically, HA is synthesized as a high-molecular weight (HMW) polymer (1000–6000 kDa) and possesses impressive hygroscopic properties, capable of retaining up to 1000 times its weight in water. Under pathological conditions, including cancer, inflammation, and tissue remodeling, HMW HA is then fragmented dynamically via hyaluronidases and reactive oxidative species into low-molecular weight (LMW) HA (10–250 kDa) and o-HA (HA oligomers, <10 kDa). Importantly, HA function varies based on its linear size; HMW HA carries anti-inflammatory, anti-proliferative, and anti-angiogenic properties, whereas LMW HA and o-HA activate immune-stimulatory and pro-angiogenic pathways (Fig. 1*A*) (40, 41, 42, 43). Intriguingly, naked mole rats possessing intrinsically decreased hyaluronidase activity and a unique variant of *Has2* that synthesizes extremely large HA (>6000 kDa) are benefited with an unusual resistance to cancer and a lifespan of at least 30 years (44). Whereas the specific mechanisms driving these size-dependent effects of HA are largely unknown, it is likely that HA size in specific contexts fosters interaction with certain binding partners and that undefined co-factors may mediate the interaction between HA and its receptors to collectively bias cellular responses in a particular direction. Together, HA and its extracellular binding partners form coordinated, substantial macromolecular structures that function as insulation and pericellular spatial buffers (40, 45).

Given its biocompatibility, nonimmunogenicity, and biodegradability, HA has been advantageous as a biomaterial used in clinical applications, such as cartilage tissue engineering, cardiac repair following myocardial infarction, drug and molecule delivery, stem cell differentiation, cosmetic moisturizing agents and fillers, and lubricants in osteoarthritic joints (46, 47). Advantageously, the viscosity and elasticity of HA solutions can be modified with its concentration and polymer size, respectively. In addition, the carboxylic acid of GlcUA or the C-6 hydroxyl group of GlcNAc can be further modified via adipic hydrazide, thiopropionyl hydrazide, tyramide, benzyl ester, glycidyl methacrylate, or bromoacetate, further widening its utility and areas of application (48). As a drug delivery biomaterial, negatively charged HA nanoparticles were engineered to deliver positively charged tumor necrosis factor–related apoptosis-inducing ligand to sites of rheumatoid arthritis in rats. Notably, packaging these ligands in HA nanocomplexes prevents its proteolysis and increases its *t*_½_ *in vivo* from 30 min to 5 days. In another application, HA was modified with thiol groups and cross-linked via disulfide linkages to form nanogels that can deliver siRNA within aqueous emulsion droplets (49).

Compared with the stability and low turnover rate of most ECM molecules, the rate of HA synthesis and degradation in the ECM is exceedingly high, as 30% of HA *in vivo* is replenished daily (50). HA is synthesized by a family of hyaluronan synthases (HASs), HAS1–3. Structurally, HAS isoenzymes are multipass transmembrane enzymes possessing large cytosolic loops wherein their glycosyltransferase catalytic activities lie. In this active site, precursors UDP-GlcNAc and UDP-GlcUA polymerize into linear HA chains and concurrently extrude through the plasma membrane via the HAS protein into the extracellular space (51). Functionally, HASs are enzymatically active at the plasma membrane. However, a substantial pool of enzymatically inactive, *N*-glycosylated HASs are localized in the endoplasmic reticulum and Golgi apparatus (52, 53).

Of all the HASs, HAS2 is the main producer of HA (51, 54). For instance, global deletion of *Has2* in mouse embryos severely stunts cardiac and vascular development and is embryonically lethal (55). In comparison, *Has1* and *Has3* knockout mice are both phenotypically viable but exhibit malformations in the retro-calcaneal bursa adjacent to the Achilles tendon (56) and vascular smooth muscle migration and neuronal development (57, 58), respectively.

## Regulation of HA synthesis via HAS2 modifications and nutrient availability

HA synthesis is modulated through the finely tuned regulation of HASs through transcriptional expression of *HAS1-3*, post-transcriptional regulation via miRNAs and long noncoding RNAs, and post-translational modifications. As a transcription factor that drives epithelial-mesenchymal transition, tumorigenesis, and metastasis, zinc finger E-box binding homeobox 1 binds to the *HAS2* promoter region and activates its transcription in breast cancer cells (59). Furthermore, transcription factors T-box transcription factor 4, retinoic acid receptor, signal transducer and activator of transcription 3, specificity proteins 1 and 3, NF-κB, and cAMP-responsive element binding protein 1 also bind to the *HAS2* promoter region and regulate its expression (60, 61, 62).

Post-transcriptionally, *HAS2* mRNA is positively regulated via its *cis*-natural antisense transcript (NAT), *HAS2-AS1*, in which exon 2 of *HAS2-AS1* exhibits partial complementarity to exon 1 of *HAS2*. This facilitates the complexing of *HAS2* mRNA with *HAS2-AS1*, stabilizing *HAS2* mRNA and increasing HAS2 expression. Of note, *O*-GlcNAcylation and acetylation around the proximal promoter region of *HAS2* further induces HAS2 expression by altering chromatin structure and increasing *HAS2-AS1* expression (63). Another post-transcriptional regulator of *HAS2*, miR-23a-3p, directly targets and inhibits *HAS2* expression, leading to the suppression of HA levels (64). Another miRNA, miR-7, suppresses *HAS2* expression indirectly via targeting epidermal growth factor receptor (EGFR) and disrupting the HA-mediated CD44-EGFR signaling pathway (65, 66).

HAS2 is regulated by a variety of post-translational modifications (67). For example, monoubiquitination of HAS2 at lysine 190 located in the glycosyltransferase-2 conserved domain is critical for its enzymatic activity (68). Nutrient conditions also heavily influence HAS2 expression and activity via post-translational regulation. Most notably, AMP kinase (AMPK), the master rheostat of cellular energy homeostasis, plays critical roles in regulating HAS2 and extracellular HA. Under nutrient-scarce conditions (*i.e.* high levels of AMP and low levels of ATP), AMPK is activated and phosphorylates HAS2 at threonine 110 located in the cytoplasmic active site. This significantly attenuates the enzymatic activity of HAS2 (69). Low-nutrient conditions, also represented by an increased NAD^+^/NADH ratio, activate an NAD^+^-dependent deacetylase, sirtuin 1. In turn, sirtuin 1 inhibits NF-κB activation and reduces *HAS2-AS1* levels. Ultimately, this decreases HAS2 expression and HA accumulation (70).

In contrast, hyperglycemic and nutrient-rich conditions wherein there are high extracellular levels of glucosamine and UDP-GlcNAc increase *O*-GlcNAcylation of HAS2, which promotes its enzymatic activity and protein stability (71). Following a similar trend, epigenetic *O*-GlcNAcylation of histones, including H3 at serine 10, in the proximal promoter region of *HAS2* promotes chromatin remodeling and induces *HAS2-AS1* and subsequently *HAS2* expression (63).

Glucose availability plays a critical role in regulating extracellular HA levels. As input levels naturally determine output yield, low environmental glucose levels result in scarcity of the UDP-sugar substrates utilized by HAS2, leading to suppression of synthesized HA. Conversely, in hyperglycemic conditions with higher levels of UDP-GlcUA and UDP-GlcNAc, extracellular HA also increase. Indeed, when depleting UDP-GlcUA levels pharmacologically via 4-methylumbelliferone (4-MU), HAS2 enzymatic activity and HA levels are suppressed (72). Specifically, 4-MU is glucuronidated by endogenous UDP-glucuronyltransferases using UDP-GlcUA as the donor, thereby decreasing a necessary precursor for HA biosynthesis. Additionally, through an unknown mechanism, 4-MU also significantly decreases *HAS2* mRNA (73, 74).

Lipid availability, as reflected by intracellular levels of prostaglandins, oxysterols, and cholesterol, also play a significant role in nutrient-associated HAS2 regulation. For instance, vasodilatory prostaglandins I_2_ and E_2_ induced *HAS2* expression via cAMP/PKA-dependent G_s_-coupled IP and EP_2_ signaling in human vascular smooth muscle cells (SMCs) (75, 76). This prostaglandin-dependent regulation of HAS2 in human saphenous vein SMCs is thought to play a role in driving the progression of atherosclerosis in saphenous vein bypass graft failure (77). Separately, oxidized low-density lipoproteins also drive atherosclerotic progression via increasing *HAS2* expression in human aortic SMCs (78, 79). Finally, the addition of cholesterol in human dermal fibroblasts increased HAS2 activity in liposomes formed by saturated phosphatidylcholine (80), and depleting cholesterol with methyl-β-cyclodextrin down-regulated *HAS2* levels via the phosphoinositide 3-kinase–Akt pathway in MCF-7 cells (81).

## Proteoglycan signaling via outside-in cues

PGs are a heterogeneous family of at least 43 protein cores with one or more covalently attached, sulfated GAG chains. Based predominantly on location, homology and protein modules, PGs are categorized into four major classes: intracellular, cell-surface, pericellular, and extracellular (5, 82). Functioning beyond mere co-receptors that present growth factors to their cognate receptors, PGs play critical roles as signaling effectors themselves. Localized predominantly at the cell surface and extracellular space, they constitute a critical component of the ECM as they oversee a myriad of processes, such as angiogenesis, morphogenesis, extracellular supramolecular assembly, migration, proliferation, cell survival, and immune regulation (8, 38, 83).

Recently, a growing body of work has emerged demonstrating the critical role of specific proteoglycans that regulate autophagy in stromal cells and their influences on physiological and cancer neovascularization (Fig. 1*B*) (38, 39). Additionally, HSPGs, HS biosynthesis, and HS-dependent signaling in the extracellular space are critical in activating autophagy in muscle and fat cells in *Drosophila* (84). Overall, the most extensively studied proteoglycans and bioactive proteoglycan fragments that signal via outside-in cues from the ECM include endorepellin, endostatin, decorin, biglycan, and lumican (Fig. 1*B*). These modulate autophagy in stromal cells as well as, in some cases, the cancer cell directly.

Endostatin, the N-terminal fragment of the HSPG collagen XVIII, is a basement membrane proteoglycan fragment that wields pro-autophagic and anti-angiogenic effects on vascular endothelial cells (85). Direct binding to VEGFR2 blocks VEGF-induced VEGFR2 activation, downstream angiogenic signaling and *VEGF* expression (86, 87). Further, endostatin also signals through α_5_β_1_ integrin on the endothelial cell surface to concurrently inhibit cell migration (88) and induce autophagy (89). Notably, through its outside-in signaling on the tumor vasculature, endostatin inhibited tumor angiogenesis in both malignant keratinocytes and mammary tumors *in vivo* (87).

Decorin is a small leucine-rich proteoglycan (82) that is active in many signaling pathways (39, 90, 91, 92, 93, 94, 95, 96). Decorin is a well-studied autophagic activator in vascular endothelial cells via binding of VEGFR2, downstream activation of AMPK, and induction of autophagic players LC3, Peg3, and Beclin 1 (12, 97, 98, 99, 100). This activation of endothelial autophagy from the extracellular matrix is considered “noncanonical” as it happens in nutrient-rich conditions and results in secretion of anti-angiogenic thrombospondin-1 (101, 102), profound catabolism of endothelial vascular endothelial growth factor A (VEGFA) (103, 104), and marked suppression of tumor angiogenesis (105). Notably, through its essential role in autophagy, decorin is also critical in sensing nutrient deprivation and modulating cardiac output functionally *in vivo* (106). Unlike endorepellin, which has no direct effects on cancer cells, decorin also binds EGFR and Met receptors expressed on tumor parenchyma and inhibits tumor growth and development through stunting proliferation and angiogenesis while activating mitophagy (107, 108, 109).

Biglycan is a small leucine-rich proteoglycan that, unlike decorin and endorepellin, promotes neovascularization in the tumor microenvironment. Mechanistically, biglycan up-regulates VEGFA expression and physically binds VEGFA, thereby indirectly promoting pro-angiogenic VEGFA-VEGFR2 signaling (110, 111). Tumor endothelial cells also hypomethylate the *BGN* promoter, epigenetically promoting biglycan expression and supporting tumor vascularization in metastatic cancers (112, 113). Separately, biglycan curtails inflammatory renal damage via triggering autophagy in human and murine peripheral blood macrophages via binding TLR4 and CD44 (9). Through TLR4 signaling, biglycan also activates autophagy in cardiomyocytes that confers protection to cardiomyocytes following ischemia and reperfusion injury (114). Although the effects of biglycan stimulating autophagy in macrophages have not been explored in a cancer model, investigation into whether biglycan aids tumor angiogenesis and growth partly via inducing autophagy in tumor-associated macrophages would contribute valuable understanding to the intricate role of extracellular matrix-evoked autophagy in cancer.

Lumican is another small leucine-rich proteoglycan that regulates collagen fibrillogenesis, embryonic development, wound healing, and tumor progression (115, 116, 117, 118, 119, 120, 121). In the context of pancreatic cancer, stromal lumican is primarily secreted from pancreatic stellate cells (122) and exerts an anti-tumor effect where high stromal levels of lumican are closely associated with decreased recurrence and increased survival following surgical resection. Downstream of binding and antagonizing EGFR in pancreatic ductal adenocarcinoma (PDAC), lumican suppresses Akt and HIF-1α signaling to inhibit glycolysis and apoptosis (123). Notably in PDAC, extracellular lumican inhibits gemcitabine-induced autophagy, a protective response to chemotherapy treatment, via down-regulating AMPK activity (124). Given the low efficacy of chemotherapeutics against pancreatic cancer, data revealing potent cytotoxicity in PDAC via lumican and gemcitabine co-treatment posit lumican as a promising ECM-derived protein therapy to sensitize cancer cells to chemotherapeutics. Separately, hypoxia-induced autophagy in pancreatic stellate cells suppress lumican production via autophagic degradation and decreased protein synthesis (122).

Endorepellin, the C-terminal domain V of perlecan, exerts its pro-autophagic and angiostatic influence on stromal endothelial cells via dual binding of VEGFR2 and α2β1 integrin, cell-surface receptors that are exclusively co-expressed in vascular endothelia. In the context of cancer, endorepellin exerts profound anti-oncogenic effects by specifically targeting the tumor neovasculature, thus reducing blood flow to the growing cancer cells (18). Specifically, when utilizing recombinant endorepellin to systemically treat human squamous carcinoma and murine Lewis lung carcinoma *in vivo*, endorepellin accumulates explicitly in the tumor vasculature, where it markedly suppresses tumor angiogenesis, metabolism, and growth while inducing intratumoral hypoxia (125).

## Perlecan and its C-terminal fragment endorepellin

Localized pericellularly in basement membranes, muscle, cartilage, and bone marrow, perlecan is a modular HSPG encoded from the highly conserved *HSPG2* gene containing 97 exons (126, 127) and a complex promoter structure (128, 129, 130). Perlecan consists of five domains in its ∼470-kDa protein core, making it one of the largest monomeric matrix molecules (131). It is critical in the development of the cardiovascular system (132), nervous system (133), and cartilage (134, 135, 136). Beyond tissue development, perlecan also regulates lipid metabolism (137, 138), angiogenesis (3, 85, 139, 140, 141, 142, 143, 144, 145), endocytosis (146), thrombosis (147), cell adhesion (148, 149), blood-brain barrier maintenance (150), and autophagy (38, 131). This comprehensive array of biofunctional properties can be attributed to specific domains in the larger parent molecule (151). For example, domain I promotes angiogenesis via its ability to sequester and present growth factors to their cognate receptors. Separately, domain V engages cell-surface receptors to inhibit angiogenesis and induce autophagy downstream (151).

Perlecan plays a fascinating role in regulating autophagy and angiogenesis that is complex and has been extensively studied in the past decade. As a whole molecule, perlecan inhibits autophagy in the slow-twitch soleus muscle of mice through activation of the mTOR complex 1 (mTORC1) pathway. Specifically, mice that lack *HSPG2* expression in muscles showed increased autophagy through mTORC1 inhibition when fasted for 24 h as demonstrated by increased levels of LC3-II, a critical component of the autophagosomal membrane, and P-AMPKα, the α subunit of the energy sensor AMPK that induces autophagy signaling downstream (85, 152), and decreased mTORC1 substrate P-p70S6K (153).

Regarding its role in modulating angiogenesis, perlecan in its entirety possesses critical pro-angiogenic capabilities. Indeed, perlecan-null mice are embryonic lethal around embryonic day 10 with perlecan-deficient embryos displaying gross cardiovascular malformations, such as irregular cardiac outflow tracts, complete transposition of great arteries, and malformed semilunar valves (154). Additionally, zebrafish with a knockdown of *Hspg2* expression show stunted angiogenic sprouting from the dorsal aorta and malformed intersegmental and subintestinal vessels (132). The mechanism fostering this pro-angiogenic phenotype lies within the three Ser-Gly-Asp sequences found within the N-terminal domain I of perlecan (155). These function as attachment sites for HS chains, which can then bind, sequester, and present a number of growth factors to their cognate receptors (156, 157, 158). These growth factors include a host of HS-binding angiokines, including progranulin, VEGFA, platelet-derived growth factor, fibroblast growth factor 2 (FGF2), FGF7, and FGF18 (159, 160, 161, 162, 163, 164). For example, perlecan promotes FGF2 interaction with its receptor to induce neovascularization in rabbit ears (165), and mice expressing a mutant perlecan containing a partial deletion of domain I in which all three HS attachment sites were annihilated show stunted angiogenesis in the cornea (166). In another example, perlecan increases VEGFA-induced activation and phosphorylation of VEGF receptor 2 (VEGFR2) (161). In regard to tumorigenesis, perlecan levels are elevated in multiple cancer cell lines and human metastatic melanoma (3, 167, 168, 169), and its ability to function as reservoir and co-receptor for angiokines enhances tumor growth and invasion (144, 162, 166, 170, 171).

In contrast, the C-terminal domain V of perlecan, referred to as endorepellin, is thought to undergo proteolytic cleavage via matrix metalloproteinases (172). This fragment adopts an antithetical functional phenotype to its larger parent molecule via activating autophagy and inhibiting angiogenesis (172, 173, 174). Coined “endorepellin” due to its ability to repel endothelial cell migration and adhesion (142), domain V of perlecan structurally consists of three laminin-like globular (LG) domains, LG1–3, separated by two EGF-like modules (82). LG3 can be further separated from the LG1/2 domains via cleavage by BMP1/Tolloid-like proteases (175) and cathepsin L (176) and has been implicated as a potential *in vivo* biomarker in a number of diseases, including renal failure, premature rupture of fetal membranes, chronic renal nephropathy, pancreatic cancer, Down syndrome, refractory cytopenia with multilineage dysplasia, IgA nephropathy, and breast cancer (177, 178, 179, 180, 181, 182, 183, 184, 185, 186, 187).

## Temporal signaling of angiostatic and autophagic endorepellin

**Figure 2:**
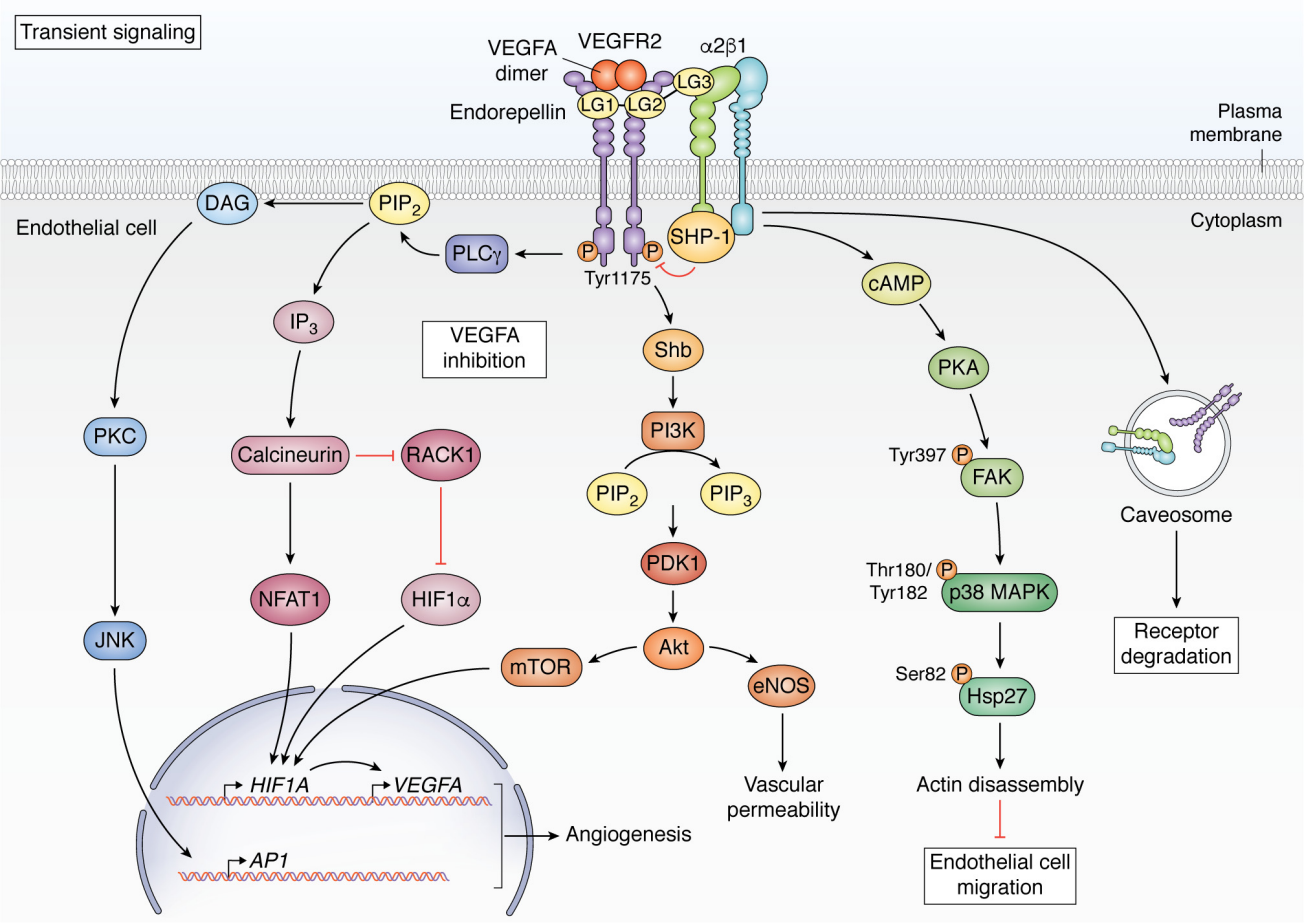
**Comprehensive schematic of transient endorepellin signaling in vascular endothelia.** The transient signaling cascade downstream of endorepellin binding begins within 30 min of endorepellin treatment in which endorepellin behaves as a dual receptor antagonist of VEGFR2 and α2β1 integrin.

Through its three LG domains, endorepellin elegantly coordinates intracellular signaling of vascular endothelial cells as a dual receptor ligand as it binds simultaneously to the major vascular RTK, VEGFR2 (188), and α2β1 integrin (189, 190) (Fig. 2). As the dual expression of VEGFR2 and α2β1 is unique to vascular endothelia and as endorepellin requires both receptors for its angiostatic activity (189), the binding and downstream effects of soluble endorepellin are specific to vascular endothelial cells. Mechanistically, LG1/2 bind the Ig_3-5_ motifs of the VEGFR2 ectodomain (191, 192), whereas LG3 concurrently binds the α2 integrin I domain of α2β1 (190, 193, 194). This “dual receptor antagonism” of endorepellin ultimately evokes angiostasis and autophagy in endothelial cells. However, endorepellin signaling through its two cognate receptors is intricate and complex. At the transient level (within 5–10 min of binding), endorepellin signals as a dual receptor antagonist and allosterically inhibits VEGFA-induced angiogenesis (189, 191, 192, 195). However, after long-term binding, this angiostatic molecule evolves into a partial agonist, where it collectively evokes autophagy and suppresses angiogenesis primarily through VEGFR2 binding (152, 196).

Transiently, endorepellin evokes internalization and down-regulation of both VEGFR2 and α2 levels within 10 min of its binding (189, 192). Second, binding of LG3 to α2β1 integrin evoked a rapid increase in cAMP levels, leading to protein kinase A (PKA) activation, phosphorylation of focal adhesion kinase, p38 mitogen-activated protein kinase, and heat shock protein 27 (Hsp27) and the anti-angiogenic disassembly of actin stress fibers and focal adhesions (190). Third, as VEGFA and endorepellin interact with VEGFR2 in separate binding pockets (Ig_2-3_ and Ig_3-5_ for VEGFA and endorepellin, respectively), endorepellin competes with VEGFA as an allosteric inhibitor as it blocks VEGFA-evoked phosphorylation of VEGFR2 at Tyr-1175 (189), *VEGFA* expression, and endothelial cell migration (189). Mechanistically, LG3 binding to α2β1 integrin evokes rapid dephosphorylation of VEGFR2 at multiple key residues, including tyrosine residue 1175 (Tyr-1175), via the physical interaction of the α2 cytoplasmic domain with and subsequent activation of Src homology 2 domain-containing protein tyrosine phosphatase 1 (SHP-1). This rapid dephosphorylation via SHP-1 that requires both VEGFR2 and α2β1 involvement diminishes *VEGFA* expression and VEGFA secretion (189). Suppression of VEGFA-induced phospho-Tyr-1175 inhibits subsequent binding and activation of phospholipase Cγ (PLCγ) (195, 197) and Src homology 2 domain-containing adaptor protein (Shb) (192), two critical downstream adaptor proteins that bind phosphorylated VEGFR2 (198, 199, 200). Via loss of PLCγ and Shb binding, endorepellin antagonizes three major angiogenic, VEGFA-evoked signaling pathways: PLCγ/PI3K/PDK1/Akt/mTOR, PLCγ/calcineurin/RACK1/NFAT1, and Shb/PKC/JNK/AP1. This widespread inhibition ultimately blocks the pro-angiogenic gene expression of *HIF1*α, *AP1*, and *NFAT1* (192) (Fig. 2).

**Figure 3:**
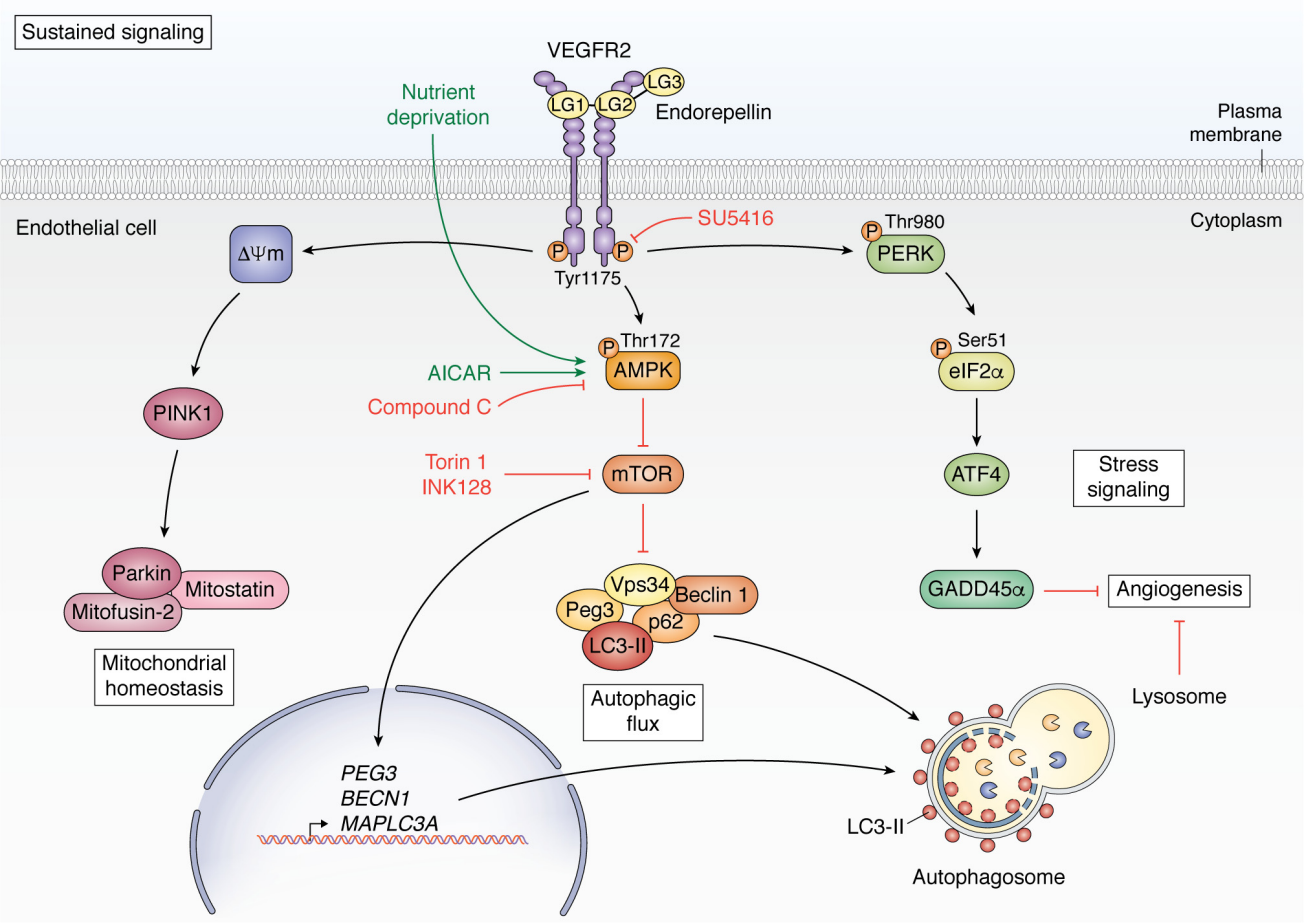
**Comprehensive schematic of sustained endorepellin signaling in vascular endothelia.** The long-term, sustained signaling cascade downstream of endorepellin binding occurs around 2–6 h in which endorepellin behaves as a partial agonist of VEGFR2 where it modulates mitochondrial homeostasis, autophagic flux, stress signaling, and angiogenesis.

In contrast, long-term endorepellin treatment concurrently evokes autophagy, stress signaling, mitochondrial depolarization, and angiostasis via an elegant signaling pathway that coordinates VEGFR2 and subsequent AMPK activation in vascular endothelia. Following 6 h of exposure, endorepellin signals as a VEGFR2 agonist phosphorylating VEGFR2 at Tyr-1175. This results in downstream activation of AMPKα at Thr-172, leading to canonical inhibition of mTOR, a potent autophagic repressor (152). Downstream, endorepellin signaling activates autophagic machinery via up-regulating protein levels and binding of autophagic markers Peg3, LC3-II, Beclin 1, and p62 as well as inducing gene expression of *PEG3*, *BECN1*, and *MAP1LC3A*. Further, this evokes formation of large, vacuolar autophagosomes in vascular endothelial cells enriched with LC3, Peg3, Vps34, p62, Beclin 1, and mTOR (152, 196). Sustained endorepellin activation of VEGFR2 also provokes the canonical PERK/eIF2α/ATF4/GADD45α stress signaling pathway (201) and mitochondrial depolarization (202) in endothelial cells. Notably, endorepellin treatment also induced decreased capillary tube formation and angiogenic inhibition *ex vivo* (152, 196). Mechanistically, endorepellin-evoked angiostasis is mediated via autophagy and stress signaling through AMPK and PERK activation, respectively (196, 201) (Fig. 3).

## HAS2, the critical link between autophagy and angiostasis

**Figure 4:**
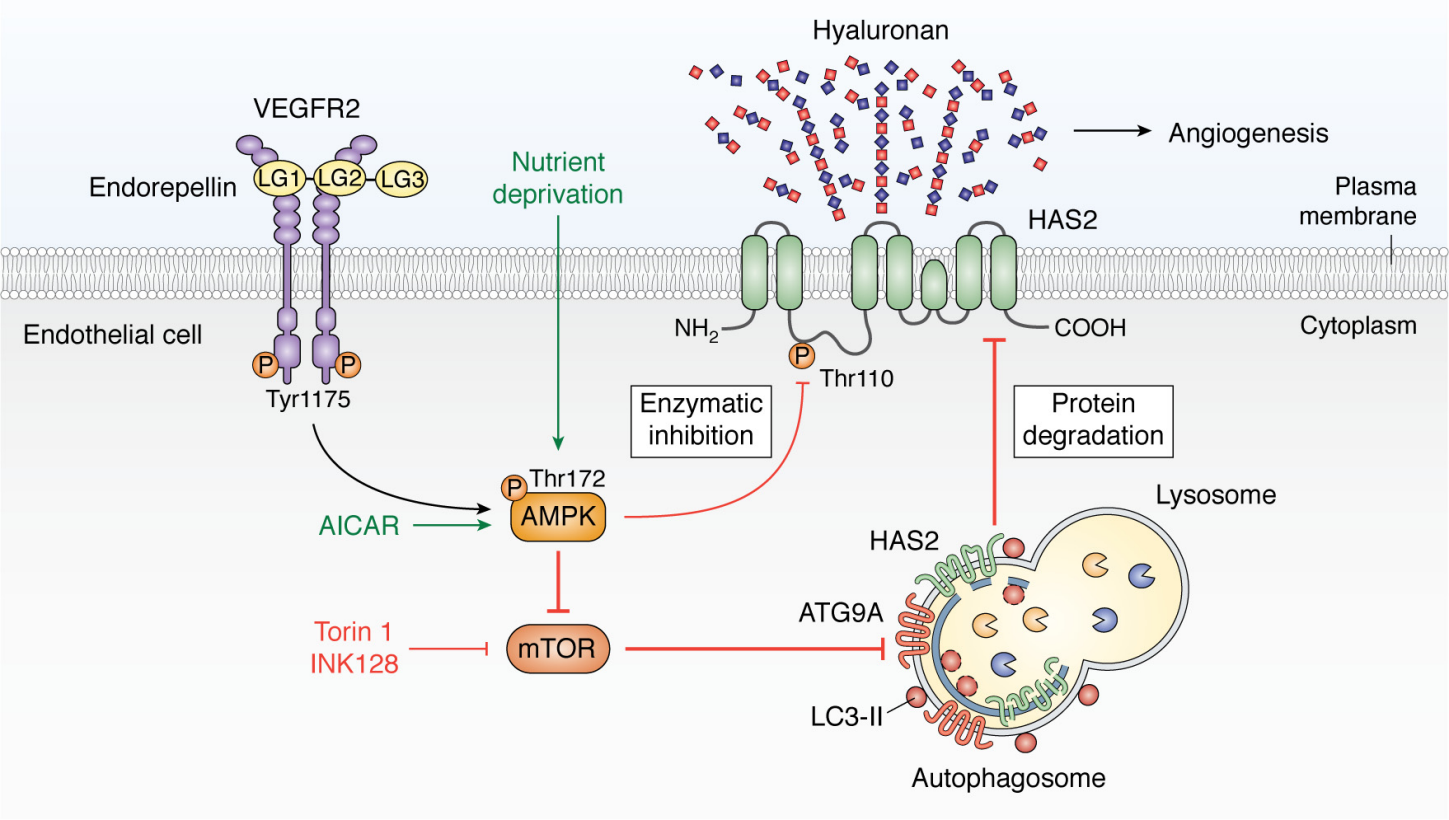
**Schematic of endorepellin-evoked catabolism of HAS2 in vascular endothelia resulting in angiostasis.** Autophagic degradation of HAS2 and suppression of HA secretion is the critical link in sustained endorepellin signaling between activation of autophagic flux and angiostasis.

Although endorepellin-induced autophagy led to downstream angiostasis (196), the mechanism linking these two intracellular processes remained a mystery. As such, the direct link connecting autophagic activation and angiostasis has been highly anticipated. Recently, we uncovered a critical and novel regulatory mechanism in which HAS2, a key producer of pro-angiogenic HA, is degraded via autophagy evoked by pro-autophagic proteoglycan fragments endorepellin and endostatin, nutrient deprivation, or mTOR inhibition. This was consistently demonstrated across a variety of cell types and species *in vitro* as well as at the organ level in heart and aorta tissue lysates of fasted mice *in vivo* (203). Notably, endorepellin induces downstream autophagic catabolism of HAS2 via VEGFR2 signaling and downstream AMPK activation under nutrient-rich conditions. Pharmacologically, AICAR, an activator of AMPK, and Torin 1 and INK128, potent inhibitors of mTOR, all phenocopy nutrient deprivation and endorepellin treatment in down-regulating cellular HAS2 levels via autophagy. This reduction of HAS2 levels translates to robust suppression of extracellular HA *in vitro* and *ex vivo* (203). Notably, marked decrease in extracellular HA via HAS2 degradation inhibits angiogenic sprouting *ex vivo* (203), effectively positing autophagic catabolism of HAS2 as a critical regulatory pathway linking autophagy to angiostasis (Fig. 4).

In support of these findings, HAS2 catalysis through the proteasome was ruled out, as inhibiting proteasomal activity via MG132 did not significantly alter total levels of HAS2 or synthesized HA (68). HAS2 regulation has also been shown to be downstream of mTOR, as mTOR activation in fibroblasts increased HAS2 levels and extracellular HA. Furthermore, inhibiting mTOR via rapamycin significantly reversed this effect (204). In fact, activating AMPK through metformin treatment has also been shown to down-regulate HA synthesis in vascular smooth muscle cells (205), further supporting an AMPK-dependent down-regulation of HAS2.

## ATG9A, a novel HAS2-binding partner

One continuing area of investigation in the study of autophagy is the source of lipid membrane that forms the growing autophagosome. Notably, the endoplasmic reticulum, mitochondria, Golgi complex, and plasma membrane have all been implicated as sources (22, 23). Among the ATG proteins, ATG9A plays a key role in transporting lipid membrane from these sources and stands apart as the only multipass transmembrane protein, a characteristic that is key to its function. Whereas its roles have not been fully elucidated, it is best defined as a shuttling protein within the ATG2-WIPI (WD-repeat protein interacting with phosphoinositides) complex that cycles between the PAS and peripheral organelles, including the *trans*-Golgi network, endosomes, and plasma membrane, to deliver lipid bilayer to the developing phagophore (25, 206, 207, 208, 209). In coordination with the core autophagic machinery, ATG9A is phosphorylated by ULK kinase core complex, a post-translational modification that is necessary to recruit LC3 to the site of autophagosome nucleation and expansion (210). During this process, ATG9A integrates into the outer autophagosomal membrane and is removed and recycled back after formation of the mature autophagosome is complete (211, 212).

Structurally, ATG9A possesses six transmembrane domains flanked by two cytosolic domains, one at each of the N- and C-terminal regions (25, 213). Similarly, HAS2 shares a structural topology akin to that of ATG9A, as it is also characterized by six transmembrane domains with both N- and C-terminal tail regions residing in the cytosol (214). Furthermore, both ATG9A and HAS2 have the ability to homodimerize. This self-interaction enables ATG9A to move anterograde to the PAS (215). Likewise, HASs may form homo- and heterodimer complexes with one another through their N-terminal domains (*i.e.* HAS1-HAS2, HAS2-HAS2, and HAS2-HAS3) (216), a structural quality critical in aiding HA synthesis (68, 69, 216). Given these shared qualities, we recently discovered that HAS2 binds ATG9A upon autophagic induction, implicating ATG9A as the protein carrier bringing HAS2 to the autophagosomal membrane. This was confirmed via enhanced pulldown of HAS2 when immunoprecipitating ATG9A in endorepellin– and mTOR inhibitor Torin 1–treated endothelial cells and increased co-localization of HAS2 and ATG9A under superresolution microscopy (203). Specifically, Torin 1–treated human umbilical vein endothelial cells demonstrated distinct vacuoles positively immunostained with ATG9A and HAS2 in contrast to vehicle-treated ones. Furthermore, this is a dynamic interaction as live-cell microscopy of porcine aortic endothelial cells transfected with RFP-ATG9A and GFP-HAS2 showed rapid formation of ATG9A- and HAS2-positive nucleating complexes within 10 and 2 min of endorepellin treatment and nutrient deprivation, respectively (203). Of note, autophagy-stimulated endothelial cells did not demonstrate HAS2 binding to either p62 or LC3-II, two well-studied proteins that selectively transport proteins to the autophagosome, effectively ruling out p62 and LC3-II involvement in facilitating HAS2 degradation (203).

## Perspective and open questions

Surrounding every cell and tissue, the ECM modulates and affects the most overarching physiological functions of the body down to myriad subcellular processes. Within this expansive system, the functional class of proteoglycans that modulates autophagy and angiogenesis via external signaling from the matrix is emerging and evolving into an exciting field in ECM biology. Certainly, with regard to cancer as well as to myriad other diseases, there is an increasing demand for drugs aimed at counteracting processes from the microenvironment, such as angiogenesis, that exacerbate disease progression. The introduction of HAS2 catalysis via autophagy as a means of regulating extracellular HA brings forth a greater understanding of the implications of autophagic induction, particularly within the realm of cancer treatment. This newfound intersection between autophagy, angiogenesis, and hyaluronan biology not only promotes our understanding hyaluronan and proteoglycan biology, but also provides an alternative strategy to curtail pathologic angiogenesis via inciting autophagy within the vasculature. Beyond HAS2-HA regulation, it is very likely that more unknown factors connecting autophagy and angiogenesis are present. Further, as the field expands, it is likely that other proteoglycans and extracellular factors will be found to modulate autophagy and angiogenesis, strengthening the tie between these two vital processes.

Notwithstanding these paramount advances in interconnecting the fields of proteoglycan signaling, HA synthesis, autophagy, and angiogenesis, there still exist large gaps and questions in our current understanding of HA dynamics that have yet to be resolved. First, what are the general mechanisms fueling size-dependent HA biology? More specifically, how does LMW HA drive angiogenic pathways at the vasculature? Second, whereas the regulatory mechanism of autophagy restricting extracellular HA focuses on HAS2, the main HAS isoform responsible for HA synthesis, are HAS1/3 also subjected to autophagic degradation? If so, do these changes in HAS1/3 levels significantly alter HA content in the matrix? Third, to further elucidate the potential role of ATG9A-HAS2 interaction in the autophagic process, is ATG9A-HAS2 binding necessary for autophagy-evoked HAS2 degradation and the subsequent decrease in secreted HA? Furthermore, does HAS2 interact with WIPI1/2 or ATG2, the two other autophagy proteins in complex with ATG9A that regulate its cycling and nucleation of LC3-positive autophagosomes (25). If so, is HAS2 itself a contributor to autophagosome development and nucleation? Fourth, the disease-driving build-up of HA in invasive breast cancer is characterized by a conglomeration of excessive hyaluronan synthase activity and HA breakdown via hyaluronidases and reactive oxygen species, resulting in an accumulation of LMW HA and HA oligosaccharides that propel tumor angiogenesis, metastasis, and inflammation and shorten overall patient survival (217). Thus, what are the therapeutic implications of HAS2 catabolism and their ensuing effects on dysregulated angiogenesis in diseases exacerbated by HA accumulation, such as cancer? As elevated levels of stromal HA transduce pro-angiogenic and tumorigenic signals in a multitude of other cancer types, including breast, prostate, ovarian, and lung (217), the therapeutic benefits of targeting HAS2 via autophagic degradation in these cancer types should be explored. Finally, independent of HAS activity and levels, there is increasing evidence that the metabolic reprogramming of cancer cells favoring glycolysis greatly up-regulates HA synthesis. Metabolic reprogramming, encompassed through the Warburg effect, drives tumorigenic growth and progression. Specifically, aggressive breast cancer demonstrates increased flux through the hexosamine biosynthetic pathway and enhanced cellular stores of GlcNAc, thereby significantly up-regulating HA synthesis and driving pro-tumorigenic signaling (218). Thus, in efforts to comprehensively target HA in the tumor microenvironment, the biology overseeing these metabolic changes in cancer and stromal metabolism adds yet another layer of complexity requiring further study.

## References

[1] Manou D., Caon I., Bouris P., Triantaphyllidou I.E., Giaroni C., Passi A., Karamanos N.K., Vigetti D., Theocharis A.D. (2019). The complex interplay between extracellular matrix and cells in tissues. Methods Mol. Biol.

[2] Ringer P., Colo G., Fässler R., Grashoff C. (2017). Sensing the mechano-chemical properties of the extracellular matrix. Matrix Biol.

[3] Cohen I.R., Murdoch A.D., Naso M.F., Marchetti D., Berd D., Iozzo R.V. (1994). Abnormal expression of perlecan proteoglycan in metastatic melanomas. Cancer Res.

[4] Iozzo R.V., Cohen I. (1993). Altered proteoglycan gene expression and the tumor stroma. Experientia.

[5] Iozzo R.V. (2005). Basement membrane proteoglycans: from cellar to ceiling. Nat. Rev. Mol. Cell Biol.

[6] Bi X., Tong C., Dockendorff A., Bancroft L., Gallagher L., Guzman G., Iozzo R.V., Augenlicht L.H., Yang W. (2008). Genetic deficiency of decorin causes intestinal tumor formation through disruption of intestinal cell maturation. Carcinogenesis.

[7] Moreth K., Iozzo R.V., Schaefer L. (2012). Small leucine-rich proteoglycans orchestrate receptor crosstalk during inflammation. Cell Cycle.

[8] Karamanos N.K., Piperigkou Z., Theocharis A.D., Watanabe H., Franchi M., Baud S., Brézillon S., Götte M., Passi A., Vigetti D., Ricard-Blum S., Sanderson R.D., Neill T., Iozzo R.V. (2018). Proteoglycan chemical diversity drives multifunctional cell regulation and therapeutics. Chem. Rev.

[9] Poluzzi C., Nastase M.V., Zeng-Brouwers J., Roedig H., Hsieh L.T., Michaelis J.B., Buhl E.M., Rezende F., Manavski Y., Bleich A., Boor P., Brandes R.P., Pfeilschifter J., Stelzer E.H.K., Münch C. (2019). Biglycan evokes autophagy in macrophages via a novel CD44/Toll-like receptor 4 signaling axis in ischemia/reperfusion injury. Kidney Int.

[10] Theocharis A.D., Gialeli C., Bouris P., Giannopoulou E., Skandalis S.S., Aletras A.J., Iozzo R.V., Karamanos N.K. (2014). Cell-matrix interactions: focus on proteoglycan-proteinase interplay and pharmacological targeting in cancer. FEBS J.

[11] Schaefer L., Tredup C., Gubbiotti M.A., Iozzo R.V. (2017). Proteoglycan neofunctions: regulation of inflammation and autophagy in cancer biology. FEBS J.

[12] Buraschi S., Neill T., Iozzo R.V. (2019). Decorin is a devouring proteoglycan: remodeling of intracellular catabolism via autophagy and mitophagy. Matrix Biol.

[13] Bouris P., Manou D., Sopaki-Valalaki A., Kolokotroni A., Moustakas A., Kapoor A., Iozzo R.V., Karamanos N.K., Theocharis A.D. (2018). Serglycin promotes breast cancer cell aggressiveness: induction of epithelial to mesenchymal transition, proteolytic activity and IL-8 signaling. Matrix Biol.

[14] Neill T., Schaefer L., Iozzo R.V. (2015). Decoding the matrix: instructive roles of proteoglycan receptors. Biochemistry.

[15] Govindaraju P., Todd L., Shetye S., Monslow J., Puré E. (2019). CD44-dependent inflammation, fibrogenesis, and collagenolysis regulates extracellular matrix remodeling and tensile strength during cutaneous wound healing. Matrix Biol.

[16] Theocharis A.D., Skandalis S.S., Gialeli C., Karamanos N.K. (2016). Extracellular matrix structure. Adv. Drug Deliv. Rev.

[17] Iozzo R.V., Gubbiotti M.A. (2018). Extracellular matrix: the driving force of mammalian diseases. Matrix Biol.

[18] Mongiat M., Buraschi S., Andreuzzi E., Neill T., Iozzo R.V. (2019). Extracellular matrix: the gatekeeper of tumor angiogenesis. Biochem. Soc. Trans.

[19] Karamanos N.K., Theocharis A.D., Neill T., Iozzo R.V. (2019). Matrix modeling and remodeling: a biological interplay regulating tissue homeostasis and diseases. Matrix Biol.

[20] Chen C., Kapoor A., Iozzo R.V. (2019). Methods for monitoring matrix-induced autophagy. Methods Mol. Biol.

[21] Neill T., Buraschi S., Kapoor A., Iozzo R.V. (2020). Proteoglycan-driven autophagy: a nutrient-independent mechanism to control intracellular catabolism. J. Histochem. Cytochem.

[22] Mizushima N., Komatsu M. (2011). Autophagy: renovation of cells and tissues. Cell.

[23] Pavel M., Rubinsztein D.C. (2017). Mammalian autophagy and the plasma membrane. FEBS J.

[24] Levine B., Kroemer G. (2008). Autophagy in the pathogenesis of disease. Cell.

[25] Li X., He S., Ma B. (2020). Autophagy and autophagy-related proteins in cancer. Mol. Cancer.

[26] Rabinowitz J.D., White E. (2010). Autophagy and metabolism. Science.

[27] Choi A.M.K., Ryter S.W., Levine B. (2013). Autophagy in human health and disease. N. Engl. J. Med.

[28] Barnard R.A., Regan D.P., Hansen R.J., Maycotte P., Thorburn A., Gustafson D.L. (2016). Autophagy inhibition delays early but not late-stage metastatic disease. J. Pharmacol. Exp. Ther.

[29] Guo J.Y., Xia B., White E. (2013). Autophagy-mediated tumor promotion. Cell.

[30] White E. (2012). Deconvoluting the context-dependent role for autophagy in cancer. Nat. Rev. Cancer.

[31] Takamura A., Komatsu M., Hara T., Sakamoto A., Kishi C., Waguri S., Eishi Y., Hino O., Tanaka K., Mizushima N. (2011). Autophagy-deficient mice develop multiple liver tumors. Genes Dev.

[32] Qu X., Yu J., Bhagat G., Furuya N., Hibshoosh H., Troxel A., Rosen J., Eskelinen E.-L., Mizushima N., Ohsumi Y., Cattoretti G., Levine B. (2003). Promotion of tumorigenesis by heterozygous disruption of the beclin 1 autophagy gene. J. Clin. Inv.

[33] Yue Z., Jin S., Yang C., Levine A.J., Heintz N. (2003). Beclin 1, an authophagy gene essential for early embryonic development, is a haploinsufficient tumor suppressor. Proc. Natl. Acad. Sci. U. S. A.

[34] Wu W.K., Coffelt S.B., Cho C.H., Wang X.J., Lee C.W., Chan F.K., Yu J., Sung J.J. (2012). The autophagic paradox in cancer therapy. Oncogene.

[35] Fung C., Lock R., Gao S., Salas E., Debnath J. (2008). Induction of autophagy during extracellular matrix detachment promotes cell survival. Mol. Biol Cell.

[36] Macintosh R.L., Timpson P., Thorburn J., Anderson K.I., Thorburn A., Ryan K.M. (2012). Inhibition of autophagy impairs tumor cell invasion in an organotypic model. Cell Cycle.

[37] Peng Y.F., Shi Y.H., Ding Z.B., Ke A.W., Gu C.Y., Hui B., Zhou J., Qiu S.J., Dai Z., Fan J. (2013). Autophagy inhibition suppresses pulmonary metastasis of HCC in mice via impairing anoikis resistance and colonization of HCC cells. Autophagy.

[38] Gubbiotti M.A., Buraschi S., Kapoor A., Iozzo R.V. (2020). Proteoglycan signaling in tumor angiogenesis and endothelial cell autophagy. Semin. Cancer Biol.

[39] Theocharis A.D., Karamanos N.K. (2019). Proteoglycans remodeling in cancer: underlying molecular mechanisms. Matrix Biol.

[40] Garantziotis S., Savani R.C. (2019). Hyaluronan biology: a complex balancing act of structure, function, location and context. Matrix Biol.

[41] Tavianatou A.G., Caon I., Franchi M., Piperigkou Z., Galesso D., Karamanos N.K. (2019). Hyaluronan: molecular size-dependent signaling and biological functions in inflammation and cancer. FEBS J.

[42] Zhang G., Wang Z., Du Z., Zhang H. (2018). mTOR regulates phase separation of PGL granules to modulate their autophagic degradation. Cell.

[43] Wang Z., Zhang H. (2019). Phase separation, transition, and autophagic degradation of proteins in development and pathogenesis. Trends Cell Biol.

[44] Tian X., Azpurua J., Hine C., Vaidya A., Myakishev-Rempel M., Ablaeva J., Mao Z., Nevo E., Gorbunova V., Seluanov A. (2013). High-molecular-mass hyaluronan mediates the cancer resistance of the naked mole rat. Nature.

[45] Huang Y., Askew E.B., Knudson C.B., Knudson W. (2016). CRISPR/Cas9 knockout of HAS2 in rat chondrosarcoma chondrocytes demonstrates the requirement of hyaluronan for aggrecan retention. Matrix Biol.

[46] Viola M., Vigetti D., Karousou E., D'Angelo M.L., Caon I., Moretto P., De Luca G., Passi A. (2015). Biology and biotechnology of hyaluronan. Glycoconj. J.

[47] Passi A., Vigetti D. (2019). Hyaluronan as tunable drug delivery system. Adv. Drug Deliv. Rev.

[48] Burdick J.A., Prestwich G.D. (2011). Hyaluronic acid hydrogels for biomedical applications. Adv. Mater.

[49] Choi K.Y., Saravanakumar G., Park J.H., Park K. (2012). Hyaluronic acid-based nanocarriers for intracellular targeting: interfacial interactions with proteins in cancer. Colloids Surf. B Biointerfaces.

[50] Bourguignon V., Flamion B. (2016). Respective roles of hyaluronidases 1 and 2 in endogenous hyaluronan turnover. FASEB J.

[51] Weigel P.H. (2015). Hyaluronan synthase: the mechanism of initiation at the reducing end and a pendulum model for polysaccharide translocation to the cell exterior. Int. J. Cell Biol.

[52] Rilla K., Siiskonen H., Spicer A.P., Hyttinen J.M., Tammi M.I., Tammi R.H. (2005). Plasma membrane residence of hyaluronan synthase is coupled to its enzymatic activity. J. Biol. Chem.

[53] Vigetti D., Genasetti A., Karousou E., Viola M., Clerici M., Bartolini B., Moretto P., De L.G., Hascall V.C., Passi A. (2009). Modulation of hyaluronan synthase activity in cellular membrane fractions. J. Biol. Chem.

[54] Passi A., Vigetti D., Buraschi S., Iozzo R.V. (2019). Dissecting the role of hyaluronan synthases in the tumor microenvironment. FEBS J.

[55] Camenisch T.D., Spicer A.P., Brehm-Gibson T., Biesterfeldt J., Augustine M.L., Calabro A., Kubalak S., Klewer S.E., McDonald J.A. (2000). Disruption of hyaluronan synthase-2 abrogates normal cardiac morphogenesis and hyaluronan-mediated transformation of epithelium to mesenchyme. J. Clin. Invest.

[56] Sikes K.J., Renner K., Li J., Grande-Allen K.J., Connell J.P., Cali V., Midura R.J., Sandy J.D., Plaas A., Wang V.M. (2018). Knockout of hyaluronan synthase 1, but not 3, impairs formation of the retrocalcaneal bursa. J. Orthop. Res.

[57] Kiene L.S., Homann S., Suvorava T., Rabausch B., Müller J., Kojda G., Kretschmer I., Twarock S., Dai G., Deenen R., Hartwig S., Lehr S., Kohrer K., Savani R.C., Grandoch M. (2016). Deletion of hyaluronan synthase 3 inhibits neointimal hyperplasia in mice. Arterioscler. Thromb. Vasc. Biol.

[58] Arranz A.M., Perkins K.L., Irie F., Lewis D.P., Hrabe J., Xiao F., Itano N., Kimata K., Hrabetova S., Yamaguchi Y. (2014). Hyaluronan deficiency due to Has3 knock-out causes altered neuronal activity and seizures via reduction in brain extracellular space. J. Neurosci.

[59] Preca B.T., Bajdak K., Mock K., Lehmann W., Sundararajan V., Bronsert P., Matzge-Ogi A., Orian-Rousseau V., Brabletz S., Brabletz T., Maurer J., Stemmler M.P. (2017). A novel ZEB1/HAS2 positive feedback loop promotes EMT in breast cancer. Oncotarget.

[60] Saavalainen K., Tammi M.I., Bowen T., Schmitz M.L., Carlberg C. (2007). Integration of the activation of the human hyaluronan synthase 2 gene promoter by common cofactors of the transcription factors retinoic acid receptor and nuclear factor kappaB. J. Biol. Chem.

[61] Vigetti D., Genasetti A., Karousou E., Viola M., Moretto P., Clerici M., Deleonibus S., De L.G., Hascall V.C., Passi A. (2010). Proinflammatory cytokines induce hyaluronan synthesis and monocyte adhesion in human endothelial cells through hyaluronan synthase 2 (HAS2) and the nuclear factor-κB (NF-κB) pathway. J. Biol. Chem.

[62] Monslow J., Williams J.D., Fraser D.J., Michael D.R., Foka P., Kift-Morgan A.P., Luo D.D., Fielding C.A., Craig K.J., Topley N., Jones S.A., Ramji D.P., Bowen T. (2006). Sp1 and Sp3 mediate constitutive transcription of the human hyaluronan synthase 2 gene. J. Biol. Chem.

[63] Vigetti D., Deleonibus S., Moretto P., Bowen T., Fischer J.W., Grandoch M., Oberhuber A., Love D.C., Hanover J.A., Cinquetti R., Karousou E., Viola M., D'Angelo M.L., Hascall V.C., De Luca G. (2014). Natural antisense transcript for hyaluronan synthase 2 (HAS2-AS1) induces transcription of HAS2 via protein *O*-GlcNAcylation. J. Biol. Chem.

[64] Röck K., Tigges J., Sass S., Schütze A., Florea A.M., Fender A.C., Theis F.J., Krutmann J., Boege F., Fritsche E., Reifenberger G., Fischer J.W. (2015). miR-23a-3p causes cellular senescence by targeting hyaluronan synthase 2: possible implication for skin aging. J. Invest. Dermatol.

[65] Midgley A.C., Bowen T., Phillips A.O., Steadman R. (2014). MicroRNA-7 inhibition rescues age-associated loss of epidermal growth factor receptor and hyaluronan-dependent differentiation in fibroblasts. Aging Cell.

[66] Midgley A.C., Rogers M., Hallett M.B., Clayton A., Bowen T., Phillips A.O., Steadman R. (2013). Transforming growth factor-β1 (TGF-β1)-stimulated fibroblast to myofibroblast differentiation is mediated by hyaluronan (HA)-facilitated epidermal growth factor receptor (EGFR) and CD44 co-localization in lipid rafts. J. Biol. Chem.

[67] Melero-Fernandez de Mera R.M., Arasu U.T., Kärnä R., Oikari S., Rilla K., Vigetti D., Passi A., Heldin P., Tammi M.I., Deen A.J. (2019). Effects of mutations in the post-translational modification sites on the trafficking of hyaluronan synthase 2 (HAS2). Matrix Biol.

[68] Karousou E., Kamiryo M., Skandalis S.S., Ruusala A., Asteriou T., Passi A., Yamashita H., Hellman U., Heldin C.H., Heldin P. (2010). The activity of hyaluronan synthase 2 is regulated by dimerization and ubiquitination. J. Biol. Chem.

[69] Vigetti D., Viola M., Karousou E., De L.G., Passi A. (2014). Metabolic control of hyaluronan synthases. Matrix Biol.

[70] Caon I., Bartolini B., Moretto P., Parnigoni A., CaravÃ E., Vitale D.L., Alaniz L., Viola M., Karousou E., De L.G., Hascall V.C., Passi A., Vigetti D. (2020). Sirtuin 1 reduces hyaluronan synthase 2 expression by inhibiting nuclear translocation of NF-Î∘B and expression of the long-noncoding RNA HAS2-AS1. J. Biol. Chem.

[71] Vigetti D., Deleonibus S., Moretto P., Karousou E., Viola M., Bartolini B., Hascall V.C., Tammi M., De L.G., Passi A. (2012). Role of UDP-*N*-acetylglucosamine (GlcNAc) and *O*-GlcNAcylation of hyaluronan synthase 2 in the control of chondroitin sulfate and hyaluronan synthesis. J. Biol. Chem.

[72] Nagy N., Kuipers H.F., Frymoyer A.R., Ishak H.D., Bollyky J.B., Wight T.N., Bollyky P.L. (2015). 4-Methylumbelliferone treatment and hyaluronan inhibition as a therapeutic strategy in inflammation, autoimmunity, and cancer. Front. Immunol.

[73] Kakizaki I., Kojima K., Takagaki K., Endo M., Kannagi R., Ito M., Maruo Y., Sato H., Yasuda T., Mita S., Kimata K., Itano N. (2004). A novel mechanism for the inhibition of hyaluronan biosynthesis by 4-methylumbelliferone. J. Biol. Chem.

[74] Kultti A., Pasonen-Seppänen S., Jauhiainen M., Rilla K.J., Karna R., Pyoria E., Tammi R.H., Tammi M.I. (2009). 4-Methylumbelliferone inhibits hyaluronan synthesis by depletion of cellular UDP-glucuronic acid and downregulation of hyaluronan synthase 2 and 3. Exp. Cell Res.

[75] Fischer J.W., Schrör K. (2007). Regulation of hyaluronan synthesis by vasodilatory prostaglandins. Implications for atherosclerosis. Thromb. Haemost.

[76] Sussmann M., Sarbia M., Meyer-Kirchrath J., Nüsing R.M., Schrör K., Fischer J.W. (2004). Induction of hyaluronic acid synthase 2 (HAS2) in human vascular smooth muscle cells by vasodilatory prostaglandins. Circ. Res.

[77] van den Boom M., Sarbia M., von Wnuck Lipinski K., Mann P., Meyer-Kirchrath J., Rauch B.H., Grabitz K., Levkau B., Schrör K., Fischer J.W. (2006). Differential regulation of hyaluronic acid synthase isoforms in human saphenous vein smooth muscle cells: possible implications for vein graft stenosis. Circ. Res.

[78] Viola M., Bartolini B., Vigetti D., Karousou E., Moretto P., Deleonibus S., Sawamura T., Wight T.N., Hascall V.C., De L.G., Passi A. (2013). Oxidized low density lipoprotein (LDL) affects hyaluronan synthesis in human aortic smooth muscle cells. J. Biol. Chem.

[79] Viola M., Karousou E., D'Angelo M.L., Caon I., De Luca G., Passi A., Vigetti D. (2015). Regulated hyaluronan synthesis by vascular cells. Int. J. Cell Biol.

[80] Ontong P., Hatada Y., Taniguchi S., Kakizaki I., Itano N. (2014). Effect of a cholesterol-rich lipid environment on the enzymatic activity of reconstituted hyaluronan synthase. Biochem. Biophys. Res. Commun.

[81] Kultti A., Kärnä R., Rilla K., Nurminen P., Koli E., Makkonen K.M., Si J., Tammi M.I., Tammi R.H. (2010). Methyl-β-cyclodextrin suppresses hyaluronan synthesis by down-regulation of hyaluronan synthase 2 through inhibition of Akt. J. Biol. Chem.

[82] Iozzo R.V., Schaefer L. (2015). Proteoglycan form and function: a comprehensive nomenclature of proteoglycans. Matrix Biol.

[83] Christensen G., Herum K.M., Lunde I.G. (2019). Sweet, yet underappreciated: proteoglycans and extracellular matrix remodeling in heart disease. Matrix Biol.

[84] Reynolds-Peterson C.E., Zhao N., Xu J., Serman T.M., Xu J., Selleck S.B. (2017). Heparan sulfate proteoglycans regulate autophagy in *Drosophila*. Autophagy.

[85] Poluzzi C., Iozzo R.V., Schaefer L. (2016). Endostatin and endorepellin: a common route of action for similar angiostatic cancer avengers. Adv. Drug Deliv. Rev.

[86] Kim Y.-M., Hwang S., Kim Y.-M., Pyun B.-J., Kim T.-Y., Lee S.-T., Gho Y.S., Kwon Y.-G. (2002). Endostatin blocks vascular endothelial growth factor-mediated signaling via direct interaction with KDR/Flk-1. J. Biol. Chem.

[87] Hajitou A., Grignet C., Devy L., Berndt S., Blacher S., Deroanne C.F., Bajou K., Fong T., Chiang Y., Foidart J.M., Noël A. (2002). The antitumoral effect of endostatin and angiostatin is associated with a down-regulation of vascular endothelial growth factor expression in tumor cells. FASEB J.

[88] Rehn M., Veikkola T., Kukk-Valdre E., Nakamura H., Ilmonen M., Lombardo C.R., Pihlajaniemi T., Alitalo K., Vuori K. (2001). Interaction of endostatin with integrins implicated in angiogenesis. Proc. Natl. Acad. Sci. U. S. A.

[89] Nguyen T.M.B., Subramanian I.V., Xiao X., Ghosh G., Nguyen P., Kelekar A., Ramakrishnan S. (2009). Endostatin induces autophagy in endothelial cells by modulating Beclin 1 and β-catenin levels. J. Cell. Mol. Med.

[90] Merline R., Moreth K., Beckmann J., Nastase M.V., Zeng-Brouwers J., Tralhão J.G., Lemarchand P., Pfeilschifter J., Schaefer R.M., Iozzo R.V., Schaefer L. (2011). Signaling by the matrix proteoglycan decorin controls inflammation and cancer through PDCD4 and microRNA-21. Sci. Signal.

[91] Ferdous Z., Wei V.M., Iozzo R.V., Höök M., Grande-Allen K.J. (2007). Decorin-transforming growth factor-β interaction regulates matrix organization and mechanical characteristics of three-dimensional collagen matrices. J. Biol. Chem.

[92] Iozzo R.V., Buraschi S., Genua M., Xu S.-Q., Solomides C.C., Peiper S.C., Gomella L.G., Owens R.T., Morrione A. (2011). Decorin antagonizes IGF receptor I (IGF-IR) function by interfering with IGF-IR activity and attenuating downstream signaling. J. Biol. Chem.

[93] Baghy K., Iozzo R.V., Kovalszky I. (2012). Decorin-TGFβ axis in hepatic fibrosis and cirrhosis. J. Histochem. Cytochem.

[94] Buraschi S., Pal N., Tyler-Rubinstein N., Owens R.T., Neill T., Iozzo R.V. (2010). Decorin antagonizes Met receptor activity and downregulates β-catenin and Myc levels. J. Biol. Chem.

[95] Buraschi S., Neill T., Owens R.T., Iniguez L.A., Purkins G., Vadigepalli R., Evans B., Schaefer L., Peiper S.C., Wang Z., Iozzo R.V. (2012). Decorin protein core affects the global gene expression profile of the tumor microenvironment in a triple-negative orthotopic breast carcinoma xenograft model. PLoS ONE.

[96] Theocharis A.D., Skandalis S.S., Neill T., Multhaupt H.A., Hubo M., Frey H., Gopal S., Gomes A., Afratis N., Lim H.C., Couchman J.R., Filmus J., Ralph D.S., Schaefer L., Iozzo R.V. (2015). Insights into the key roles of proteoglycans in breast cancer biology and translational medicine. Biochim. Biophys. Acta.

[97] Buraschi S., Neill T., Goyal A., Poluzzi C., Smythies J., Owens R.T., Schaefer L., Torres A., Iozzo R.V. (2013). Decorin causes autophagy in endothelial cells via Peg3. Proc. Natl. Acad. Sci. U. S. A.

[98] Gubbiotti M.A., Iozzo R.V. (2015). Proteoglycans regulate autophagy via outside-in signaling: an emerging new concept. Matrix Biol.

[99] Neill T., Torres A.T., Buraschi S., Iozzo R.V. (2013). Decorin has an appetite for endothelial cell autophagy. Autophagy.

[100] Neill T., Schaefer L., Iozzo R.V. (2012). Decorin, a guardian from the matrix. Am. J. Pathol.

[101] Neill T., Jones H.R., Crane-Smith Z., Owens R.T., Schaefer L., Iozzo R.V. (2013). Decorin induces rapid secretion of thrombospondin-1 in basal breast carcinoma cells via inhibition of Ras homolog gene family, member A/Rho-associated coiled-coil containing protein kinase 1. FEBS J.

[102] Torres A., Gubbiotti M.A., Iozzo R.V. (2017). Decorin-inducible Peg3 evokes Beclin 1-mediated autophagy and thrombospondin 1-mediated angiostasis. J. Biol. Chem.

[103] Neill T., Chen C.G., Buraschi S., Iozzo R.V. (2020). Catabolic degradation of endothelial VEGFA via autophagy. J. Biol. Chem.

[104] Neill T., Painter H., Buraschi S., Owens R.T., Lisanti M.P., Schaefer L., Iozzo R.V. (2012). Decorin antagonizes the angiogenic network: concurrent inhibition of Met, hypoxia inducible factor-1α and vascular endothelial growth factor A and induction of thrombospondin-1 and TIMP3. J. Biol. Chem.

[105] Neill T., Schaefer L., Iozzo R.V. (2016). Decorin as a multivalent therapeutic agent against cancer. Adv. Drug Deliv. Rev.

[106] Gubbiotti M.A., Seifert E., Rodeck U., Hoek J.B., Iozzo R.V. (2018). Metabolic reprogramming of murine cardiomyocytes during autophagy requires the extracellular nutrient sensor decorin. J. Biol. Chem.

[107] Neill T., Torres A., Buraschi S., Owens R.T., Hoek J., Baffa R., Iozzo R.V. (2014). Decorin induces mitophagy in breast carcinoma cells via peroxisome proliferator-activated receptor γ coactivator-1α (PGC-1α) and mitostatin. J. Biol. Chem.

[108] Iozzo R.V., Moscatello D., McQuillan D.J., Eichstetter I. (1999). Decorin is a biological ligand for the epidermal growth factor receptor. J. Biol. Chem.

[109] Goldoni S., Humphries A., Nyström A., Sattar S., Owens R.T., McQuillan D.J., Ireton K., Iozzo R.V. (2009). Decorin is a novel antagonistic ligand of the Met receptor. J. Cell Biol.

[110] Berendsen A.D., Pinnow E.L., Maeda A., Brown A.C., McCartney-Francis N., Kram V., Owens R.T., Robey P.G., Holmbeck K., de Castro L.F., Kilts T.M., Young M.F. (2014). Biglycan modulates angiogenesis and bone formation during fracture healing. Matrix Biol.

[111] Xing X., Gu X., Ma T., Ye H. (2015). Biglycan up-regulated vascular endothelial growth factor (VEGF) expression and promoted angiogenesis in colon cancer. Tumour Biol.

[112] Maishi N., Ohba Y., Akiyama K., Ohga N., Hamada J., Nagao-Kitamoto H., Alam M.T., Yamamoto K., Kawamoto T., Inoue N., Taketomi A., Shindoh M., Hida Y., Hida K. (2016). Tumour endothelial cells in high metastatic tumours promote metastasis via epigenetic dysregulation of biglycan. Sci. Rep.

[113] Maishi N., Hida K. (2017). Tumor endothelial cells accelerate tumor metastasis. Cancer Sci.

[114] Gáspár R., Pipicz M., Hawchar F., Kovács D., Djirackor L., Görbe A., Varga Z.V., Kiricsi M., Petrovski G., Gácser A., Csonka C., Csont T. (2016). The cytoprotective effect of biglycan core protein involves Toll-like receptor 4 signaling in cardiomyocytes. J. Mol. Cell Cardiol.

[115] Neame P.J., Kay C.J., McQuillan D.J., Beales M.P., Hassell J.R. (2000). Independent modulation of collagen fibrillogenesis by decorin and lumican. Cell Mol. Life Sci.

[116] Vuillermoz B., Khoruzhenko A., D'Onofrio M.-F., Ramont L., Venteo L., Perreau C., Antonicelli F., Maquart F.-X., Wegrowski Y. (2004). The small leucine-rich proteoglycan lumican inhibits melanoma progression. Exp. Cell Res.

[117] Meij J.T.A., Carlson E.C., Wang L., Liu C.-Y., Jester J.V., Birk D.E., Kao W.W.Y. (2007). Targeted expression of a lumican transgene rescues corneal deficiencies in lumican-null mice. Mol. Vis.

[118] Brézillon S., Venteo L., Ramont L., D'Onofrio M.-F., Perreau C., Pluot M., Maquart F.-X., Wegrowski Y. (2007). Expression of lumican, a small leucine-rich proteoglycan with antitumour activity, in human malignant melanoma. Clin. Exp. Dermatol.

[119] Albig A.R., Roy T.G., Becenti D.J., Schiemann W.P. (2007). Transcriptome analysis of endothelial cell gene expression induced by growth on Matrigel matrices: identification and characterization of MAGP-2 and lumican as novel regulators of angiogenesis. Angiogenesis.

[120] Chakravarti S. (2002). Functions of lumican and fibromodulin: lessons from knockout mice. Glycoconj. J.

[121] Coulson-Thomas V.J., Coulson-Thomas Y.M., Gesteira T.F., Andrade de Paula C.A., Carneiro C.R., Ortiz V., Toma L., Kao W.W., Nader H.B. (2013). Lumican expression, localization and antitumor activity in prostate cancer. Exp. Cell Res.

[122] Sarcar B., Li X., Fleming J.B. (2019). Hypoxia-induced autophagy degrades stromal lumican into tumor microenvironment of pancreatic ductal adenocarcinoma: a mini-review. J. Cancer Treatment Diagn.

[123] Li X., Truty M.A., Kang Y., Chopin-Laly X., Zhang R., Roife D., Chatterjee D., Lin E., Thomas R.M., Wang H., Katz M.H., Fleming J.B. (2014). Extracellular lumican inhibits pancreatic cancer cell growth and is associated with prolonged survival after surgery. Clin. Cancer Res.

[124] Li X., Roife D., Kang Y., Dai B., Pratt M., Fleming J.B. (2016). Extracellular lumican augments cytotoxicity of chemotherapy in pancreatic ductal adenocarcinoma cells via autophagy inhibition. Oncogene.

[125] Bix G., Castello R., Burrows M., Zoeller J.J., Weech M., Iozzo R.A., Cardi C., Thakur M.T., Barker C.A., Camphausen K.C., Iozzo R.V. (2006). Endorepellin *in vivo*: targeting the tumor vasculature and retarding cancer growth and metabolism. J. Natl. Cancer Inst.

[126] Noonan D.M., Fulle A., Valente P., Cai S., Horigan E., Sasaki M., Yamada Y., Hassell J.R. (1991). The complete sequence of perlecan, a basement membrane heparan sulfate proteoglycan, reveals extensive similarity with laminin A chain, low density lipoprotein-receptor, and the neural cell adhesion molecule. J. Biol. Chem.

[127] Thomas K.R., Capecchi M.R. (1990). Targeted disruption of the murine *int-1* proto-oncogene resulting in severe abnormalities in midbrain and cerebellar development. Nature.

[128] Iozzo R.V., Pillarisetti J., Sharma B., Murdoch A.D., Danielson K.G., Uitto J., Mauviel A. (1997). Structural and functional characterization of the human perlecan gene promoter: transcriptional activation by transforming factor-β via a nuclear factor 1-binding element. J. Biol. Chem.

[129] Iozzo R.V. (1998). Matrix proteoglycans: from molecular design to cellular function. Annu. Rev. Biochem.

[130] Farach-Carson M.C., Warren C.R., Harrington D.A., Carson D.D. (2014). Border patrol: insights into the unique role of perlecan/heparan sulfate proteoglycan 2 at cell and tissue borders. Matrix Biol.

[131] Martinez J.R., Dhawan A., Farach-Carson M.C. (2018). Modular proteoglycan perlecan/HSPG2: mutations, phenotypes, and functions. Genes (Basel).

[132] Zoeller J.J., McQuillan A., Whitelock J., Ho S.-Y., Iozzo R.V. (2008). A central function for perlecan in skeletal muscle and cardiovascular development. J. Cell Biol.

[133] Colombelli C., Palmisano M., Eshed-Eisenbach Y., Zambroni D., Pavoni E., Ferri C., Saccucci S., Nicole S., Soininen R., McKee K.K., Yurchenco P.D., Peles E., Wrabetz L., Feltri M.L. (2015). Perlecan is recruited by dystroglycan to nodes of Ranvier and binds the clustering molecule gliomedin. J. Cell Biol.

[134] Hassell J.R., Yamada Y., Arikawa-Hirasawa E. (2002). Role of perlecan in skeletal development and diseases. Glycoconj. J.

[135] Jochmann K., Bachvarova V., Vortkamp A. (2014). Heparan sulfate as a regulator of endochondral ossification and osteochondroma development. Matrix Biol.

[136] Wilusz R.E., Sanchez-Adams J., Guilak F. (2014). The structure and function of the pericellular matrix of articular cartilage. Matrix Biol.

[137] Fuki I., Iozzo R.V., Williams K.J. (2000). Perlecan heparan sulfate proteoglycan. A novel receptor that mediates a distinct pathway for ligand catabolism. J. Biol. Chem.

[138] Yamashita Y., Nakada S., Yoshihara T., Nara T., Furuya N., Miida T., Hattori N., Arikawa-Hirasawa E. (2018). Perlecan, a heparan sulfate proteoglycan, regulates systemic metabolism with dynamic changes in adipose tissue and skeletal muscle. Sci. Rep.

[139] McCarthy K.J. (2015). The basement membrane proteoglycans perlecan and agrin: something old, something new. Curr. Top. Membr.

[140] Iozzo R.V. (1988). Proteoglycans and neoplasia. Cancer Metastasis Rev.

[141] Mathiak M., Yenisey C., Grant D.S., Sharma B., Iozzo R.V. (1997). A role for perlecan in the suppression of growth and invasion in fibrosarcoma cells. Cancer Res.

[142] Mongiat M., Sweeney S., San Antonio J.D., Fu J., Iozzo R.V. (2003). Endorepellin, a novel inhibitor of angiogenesis derived from the C terminus of perlecan. J. Biol. Chem.

[143] Iozzo R.V., San Antonio J.D. (2001). Heparan sulfate proteoglycans: heavy hitters in the angiogenesis arena. J. Clin. Invest.

[144] Iozzo R.V., Zoeller J.J., Nyström A. (2009). Basement membrane proteoglycans: modulators par excellence of cancer growth and angiogenesis. Mol. Cells.

[145] Iozzo R.V., Sanderson R.D. (2011). Proteoglycans in cancer biology, tumour microenvironment and angiogenesis. J. Cell. Mol. Med.

[146] Christianson H.C., Belting M. (2014). Heparan sulfate proteoglycan as a cell-surface endocytosis receptor. Matrix Biol.

[147] Nugent M.A., Nugent H.M., Iozzo R.V., Sanchack K., Edelman E.R. (2000). Perlecan is required to inhibit thrombosis after deep vascular injury and contributes to endothelial cell-mediated inhibition of intimal hyperplasia. Proc. Natl. Acad. Sci. U. S. A.

[148] Lord M.S., Chuang C.Y., Melrose J., Davies M.J., Iozzo R.V., Whitelock J.M. (2014). The role of vascular-derived perlecan in modulating cell adhesion, proliferation and growth factor signaling. Matrix Biol.

[149] Whitelock J.M., Graham L.D., Melrose J., Murdoch A.D., Iozzo R.V., Underwood P.A. (1999). Human perlecan immunopurified from different endothelial cell sources has different adhesive properties for vascular cells. Matrix Biol.

[150] Nakamura K., Ikeuchi T., Nara K., Rhodes C.S., Zhang P., Chiba Y., Kazuno S., Miura Y., Ago T., Arikawa-Hirasawa E., Mukouyama Y.S., Yamada Y. (2019). Perlecan regulates pericyte dynamics in the maintenance and repair of the blood-brain barrier. J. Cell Biol.

[151] Gubbiotti M.A., Neill T., Iozzo R.V. (2017). A current view of perlecan in physiology and pathology: a mosaic of functions. Matrix Biol.

[152] Poluzzi C., Casulli J., Goyal A., Mercer T.J., Neill T., Iozzo R.V. (2014). Endorepellin evokes autophagy in endothelial cells. J. Biol. Chem.

[153] Ning L., Xu Z., Furuya N., Nonaka R., Yamada Y., Arikawa-Hirasawa E. (2015). Perlecan inhibits autophagy to maintain muscle homeostasis in mouse soleus muscle. Matrix Biol.

[154] Costell M., Carmona R., Gustafsson E., González-Iriarte M., Fässler R., Muñoz-Chápuli R. (2002). Hyperplastic conotruncal endocardial cushions and transposition of great arteries in perlecan-null mice. Circ. Res.

[155] Murdoch A.D., Dodge G.R., Cohen I., Tuan R.S., Iozzo R.V. (1992). Primary structure of the human heparan sulfate proteoglycan from basement membrane (HSPG2/perlecan). A chimeric molecule with multiple domains homologous to the low density lipoprotein receptor, laminin, neural cell adhesion molecules, and epidermal growth factor. J. Biol. Chem.

[156] Weihua X., Kolla V., Kalvakolanu D.V. (1997). Modulation of interferon action by retinoids: induction of murine STAT1 gene expression by retinoic acid. J. Biol. Chem.

[157] Mongiat M., Otto J., Oldershaw R., Ferrer F., Sato J.D., Iozzo R.V. (2001). Fibroblast growth factor-binding protein is a novel partner for perlecan protein core. J. Biol. Chem.

[158] Muthusamy A., Cooper C.R., Gomes R.R. (2010). Soluble perlecan domain I enhances vascular endothelial growth factor-165 activity and receptor phosphorylation in human bone marrow endothelial cells. BMC Biochem.

[159] Mongiat M., Taylor K., Otto J., Aho S., Uitto J., Whitelock J., Iozzo R.V. (2000). The protein core of the proteoglycan perlecan binds specifically to fibroblast growth factor-7. J. Biol. Chem.

[160] Smith S.M.L., West L.A., Govindraj P., Zhang X., Ornitz D.M., Hassell J.R. (2007). Heparan and chondroitin sulfate on growth plate perlecan mediate binding and delivery of FGF-2 to FGF receptors. Matrix Biol.

[161] Zoeller J.J., Whitelock J., Iozzo R.V. (2009). Perlecan regulates developmental angiogenesis by modulating the VEGF-VEGFR2 axis. Matrix Biol.

[162] Gonzalez E.M., Mongiat M., Slater S.J., Baffa R., Iozzo R.V. (2003). A novel interaction between perlecan protein core and progranulin: Potential effects on tumor growth. J. Biol. Chem.

[163] Tanimoto R., Palladino C., Xu S.Q., Buraschi S., Neill T., Gomella L.G., Peiper S.C., Belfiore A., Iozzo R.V., Morrione A. (2017). The perlecan-interacting growth factor progranulin regulates ubiquitination, sorting, and lysosomal degradation of sortilin. Matrix Biol.

[164] Chuang C.Y., Lord M.S., Melrose J., Rees M.D., Knox S.M., Freeman C., Iozzo R.V., Whitelock J. (2010). Heparan sulfate-dependent signaling of fibroblast growth growth factor 18 by chondrocyte-derived perlecan. Biochemistry.

[165] Aviezer D., Hecht D., Safran M., Eisinger M., David G., Yayon A. (1994). Perlecan, basal lamina proteoglycan, promotes basic fibroblast growth factor-receptor binding, mitogenesis, and angiogenesis. Cell.

[166] Zhou Z., Wang J., Cao R., Morita H., Soininen R., Chan K.M., Liu B., Cao Y., Tryggvason K. (2004). Impaired angiogenesis, delayed wound healing and retarded tumor growth in perlecan heparan sulfate-deficient mice. Cancer Res.

[167] Iozzo R.V. (1984). Biosynthesis of heparan sulfate proteoglycan by human colon carcinoma cells and its localization at the cell surface. J. Cell Biol.

[168] Tapanadechopone P., Tumova S., Jiang X., Couchman J.R. (2001). Epidermal transformation leads to increased perlecan synthesis with heparin-binding-growth-factor affinity. Biochem. J.

[169] Iozzo R.V., Cohen I.R., Grässel S., Murdoch A.D. (1994). The biology of perlecan: the multifaceted heparan sulphate proteoglycan of basement membranes and pericellular matrices. Biochem. J.

[170] Aviezer D., Iozzo R.V., Noonan D.M., Yayon A. (1997). Suppression of autocrine and paracrine functions of basic fibroblast growth factor by stable expression of perlecan antisense cDNA. Mol. Cell Biol.

[171] Adatia R., Albini A., Carlone S., Giunciuglio D., Benelli R., Santi L., Noonan D.M. (1997). Suppression of invasive behavior of melanoma cells by stable expression of anti-sense perlecan cDNA. Ann. Oncol.

[172] Birk D.E., Hahn R.A., Linsenmayer C., Zycband E.I. (1996). Characterization of collagen fibril segments from chicken embryo cornea, dermis and tendon. Matrix Biol.

[173] Bix G., Iozzo R.V. (2005). Matrix revolutions: “tails” of basement-membrane components with angiostatic functions. Trends Cell Biol.

[174] Bix G., Iozzo R.V. (2008). Novel interactions of perlecan: unraveling perlecan's role in angiogenesis. Microsc. Res.

[175] Gonzalez E.M., Reed C.C., Bix G., Fu J., Zhang Y., Gopalakrishnan B., Greenspan D.S., Iozzo R.V. (2005). BMP-1/Tolloid-like metalloproteases process endorepellin, the angiostatic C-terminal fragment of perlecan. J. Biol. Chem.

[176] Cailhier J.-F., Sirois I., Raymond M.-A., Lepage S., Laplante P., Brassard N., Prat A., Iozzo R.V., Pshezhetsky A.V., Hébert M.-J. (2008). Caspase-3 activation triggers extracellular release of cathepsin L and endorepellin proteolysis. J. Biol. Chem.

[177] Parker T.J., Sampson D.L., Broszczak D., Chng Y.L., Carter S.L., Leavesley D.I., Parker A.W., Upton Z. (2012). A fragment of the LG3 peptide of endorepellin is present in the urine of physically active mining workers: a potential marker of physical activity. PLoS ONE.

[178] Oda O., Shinzato T., Ohbayashi K., Takai I., Kunimatsu M., Maeda K., Yamanaka N. (1996). Purification and characterization of perlecan fragment in urine of end-stage renal failure patients. Clin. Chim. Acta.

[179] Vuadens F., Benay C., Crettaz D., Gallot D., Sapin V., Schneider P., Bienvenut W.-V., Lémery D., Quadroni M., Dastugue B., Tissot J.-D. (2003). Identification of biologic markers of the premature rupture of fetal membranes: proteomic approach. Proteomics.

[180] O'Riordan E., Orlova T.N., Mendelev N., Patschan D., Kemp R., Chander P.N., Hu R., Hao G., Gross S.S., Iozzo R.V., Delaney V., Goligorsky M.S. (2008). Urinary proteomic analysis of chronic renal allograft nephropathy. Proteomics Clin. Appl.

[181] Mauri P., Scarpa A., Nascimbeni A.C., Benazzi L., Parmagnani E., Mafficini A., Della Peruta M., Bassi C., Miyazaki K., Sorio C. (2005). Identification of proteins released by pancreatic cancer cells by multidimensional protein identification technology: a strategy for identification of novel cancer markers. FASEB J.

[182] Grønborg M., Kristiansen T.Z., Iwahori A., Chang R., Reddy R., Sato N., Molina H., Jensen O.N., Hruban R.H., Goggins M.G., Maitra A., Pandey A. (2006). Biomarker discovery from pancreatic cancer secretome using a differential proteomic approach. Mol. Cell. Proteomics.

[183] Tsangaris G.T., Karamessinis P., Kolialexi A., Garbis S.D., Antsaklis A., Mavrou A., Fountoulakis M. (2006). Proteomic analysis of amniotic fluid in pregnancies with Down syndrome. Proteomics.

[184] Aspinall-O'Dea M., Costello E. (2007). The pancreatic cancer proteome-recent advances and future promise. Proteomics Clin. Appl.

[185] Májek P., Reicheltová Z., Suttnar J., Čermák J., Dyr J.E. (2011). Plasma proteome changes associated with refractory cytopenia with multilineage dysplasia. Proteome Sci.

[186] Surin B., Sachon E., Rougier J.-P., Steverlynck C., Garreau C., Lelongt B., Ronco P., Piedagnel R. (2013). LG3 fragment of endorepellin is a possible biomarker of severity in IgA nephropathy. Proteomics.

[187] Chang J.W., Kang U.-B., Kim D.H., Yi J.K., Lee J.W., Noh D.-Y., Lee C., Yu M.-H. (2008). Identification of circulating endorepellin LG3 fragment: potential use as a serological biomarker for breast cancer. Proteomics Clin. Appl.

[188] Simons M., Gordon E., Claesson-Welsh L. (2016). Mechanisms and regulation of endothelial VEGF receptor signalling. Nat. Rev. Mol. Cell Biol.

[189] Goyal A., Pal N., Concannon M., Paul M., Doran M., Poluzzi C., Sekiguchi K., Whitelock J.M., Neill T., Iozzo R.V. (2011). Endorepellin, the angiostatic module of perlecan, interacts with both the α2β1 integrin and vascular endothelial growth factor receptor 2 (VEGFR2). J. Biol. Chem.

[190] Bix G., Fu J., Gonzalez E., Macro L., Barker A., Campbell S., Zutter M.M., Santoro S.A., Kim J.K., Höök M., Reed C.C., Iozzo R.V. (2004). Endorepellin causes endothelial cell disassembly of actin cytoskeleton and focal adhesions through the α2β1 integrin. J. Cell Biol.

[191] Willis C.D., Poluzzi C., Mongiat M., Iozzo R.V. (2013). Endorepellin laminin-like globular repeat 1/2 domains bind Ig3-5 of vascular endothelial growth factor (VEGF) receptor 2 and block pro-angiogenic signaling by VEGFA in endothelial cells. FEBS J.

[192] Goyal A., Poluzzi C., Willis A.C., Smythies J., Shellard A., Neill T., Iozzo R.V. (2012). Endorepellin affects angiogenesis by antagonizing diverse VEGFR2- evoked signaling pathways: transcriptional repression of HIF-1a and VEGFA and concurrent inhibition of NFAT1 activation. J. Biol. Chem.

[193] Bix G., Iozzo R.A., Woodall B., Burrows M., McQuillan A., Campbell S., Fields G.B., Iozzo R.V. (2007). Endorepellin, the C-terminal angiostatic module of perlecan, enhances collagen-platelet responses via the α2β1 integrin receptor. Blood.

[194] Woodall B.P., Nyström A., Iozzo R.A., Eble J.A., Niland S., Krieg T., Eckes B., Pozzi A., Iozzo R.V. (2008). Integrin α2β1 is the required receptor for endorepellin angiostatic activity. J. Biol. Chem.

[195] Nyström A., Shaik Z.P., Gullberg D., Krieg T., Eckes B., Zent R., Pozzi A., Iozzo R.V. (2009). Role of tyrosine phosphatase SHP-1 in the mechanism of endorepellin angiostatic activity. Blood.

[196] Goyal A., Gubbiotti M.A., Chery D.R., Han L., Iozzo R.V. (2016). Endorepellin-evoked autophagy contributes to angiostasis. J. Biol. Chem.

[197] Bhattacharya R., Kwon J., Wang E., Mukherjee P., Mukhopadhyay D. (2008). Src homology 2 (SH2) domain containing protein tyrosine phosphatase-1 (SHP-1) dephosphorylates VEGF receptor-2 and attenuates endothelial DNA synthesis, but not migration. J. Mol. Signal.

[198] Koch S., Tugues S., Li X., Gualandi L., Claesson-Welsh L. (2011). Signal transduction by vascular endothelial growth factor receptors. Biochem. J.

[199] Holmes K., Roberts O.L., Thomas A.M., Cross M.J. (2007). Vascular endothelial growth factor receptor-2: structure, function, intracellular signalling and therapeutic inhibition. Cell. Signal.

[200] Olsson A.-K., Dimberg A., Kreuger J., Claesson-Welsh L. (2006). VEGF receptor signalling—in control of vascular function. Nat. Rev. Mol. Cell Biol.

[201] Kapoor A., Chen C.G., Iozzo R.V. (2020). Endorepellin evokes an angiostatic stress signaling cascade in endothelial cells. J. Biol. Chem.

[202] Neill T., Andreuzzi E., Wang Z.-X., Peiper S.C., Mongiat M., Iozzo R.V. (2018). Endorepellin remodels the endothelial transcriptome toward a pro-autophagic and pro-mitophagic gene signature. J. Biol. Chem.

[203] Chen C.G., Gubbiotti M.A., Kapoor A., Han X., Yu Y., Linhardt R.J., Iozzo R.V. (2020). Autophagic degradation of HAS2 in endothelial cells: A novel mechanism to regulate angiogenesis. Matrix Biol.

[204] Qin J., Berdyshev E., Poirer C., Schwartz N.B., Dawson G. (2012). Neutral sphingomyelinase 2 deficiency increases hyaluronan synthesis by up-regulation of Hyaluronan synthase 2 through decreased ceramide production and activation of Akt. J. Biol. Chem.

[205] Sainio A., Takabe P., Oikari S., Salomäki-Myftari H., Koulu M., Soderstrom M., Pasonen-Seppanen S., Järveläinen H. (2020). Metformin decreases hyaluronan synthesis by vascular smooth muscle cells. J. Investig. Med.

[206] Mattera R., Park S.Y., De P.R., Guardia C.M., Bonifacino J.S. (2017). AP-4 mediates export of ATG9A from the *trans*-Golgi network to promote autophagosome formation. Proc. Natl. Acad. Sci. U. S. A.

[207] Zhuang X., Chung K.P., Cui Y., Lin W., Gao C., Kang B.H., Jiang L. (2017). ATG9 regulates autophagosome progression from the endoplasmic reticulum in *Arabidopsis*. Proc. Natl. Acad. Sci. U. S. A.

[208] Ivankovic D., Drew J., Lesept F., White I.J., López D.G., Tooze S.A., Kittler J.T. (2020). Axonal autophagosome maturation defect through failure of ATG9A sorting underpins pathology in AP-4 deficiency syndrome. Autophagy.

[209] Ungermann C., Reggiori F. (2018). Atg9 proteins, not so different after all. Autophagy.

[210] Papinski D., Schuschnig M., Reiter W., Wilhelm L., Barnes C.A., Maiolica A., Hansmann I., Pfaffenwimmer T., Kijanska M., Stoffel I., Lee S.S., Brezovich A., Lou J.H., Turk B.E., Aebersold R. (2014). Early steps in autophagy depend on direct phosphorylation of Atg9 by the Atg1 kinase. Mol. Cell.

[211] Yamamoto H., Kakuta S., Watanabe T.M., Kitamura A., Sekito T., Kondo-Kakuta C., Ichikawa R., Kinjo M., Ohsumi Y. (2012). Atg9 vesicles are an important membrane source during early steps of autophagosome formation. J. Cell Biol.

[212] Reggiori F., Tooze S.A. (2012). Autophagy regulation through Atg9 traffic. J. Cell Biol.

[213] Young A.R., Chan E.Y., Hu X.W., Köchl R., Crawshaw S.G., High S., Hailey D.W., Lippincott-Schwartz J., Tooze S.A. (2006). Starvation and ULK1-dependent cycling of mammalian Atg9 between the TGN and endosomes. J. Cell Sci.

[214] Weigel P.H., DeAngelis P.L. (2007). Hyaluronan synthases: a decade-plus of novel glycosyltransferases. J. Biol. Chem.

[215] He C., Baba M., Cao Y., Klionsky D.J. (2008). Self-interaction is critical for Atg9 transport and function at the phagophore assembly site during autophagy. Mol. Biol. Cell.

[216] Bart G., Vico N.O., Hassinen A., Pujol F.M., Deen A.J., Ruusala A., Tammi R.H., Squire A., Heldin P., Kellokumpu S., Tammi M.I. (2015). Fluorescence resonance energy transfer (FRET) and proximity ligation assays reveal functionally relevant homo- and heteromeric complexes among hyaluronan synthases HAS1, HAS2, and HAS3. J. Biol. Chem.

[217] Chanmee T., Ontong P., Itano N. (2016). Hyaluronan: a modulator of the tumor microenvironment. Cancer Lett.

[218] Chokchaitaweesuk C., Kobayashi T., Izumikawa T., Itano N. (2019). Enhanced hexosamine metabolism drives metabolic and signaling networks involving hyaluronan production and *O*-GlcNAcylation to exacerbate breast cancer. Cell Death Dis.

